# Recent Drug Development in the Woodchuck Model of Chronic Hepatitis B

**DOI:** 10.3390/v14081711

**Published:** 2022-08-03

**Authors:** Manasa Suresh, Stephan Menne

**Affiliations:** Georgetown University Medical Center, Washington, DC 20057, USA; ms3687@georgetown.edu

**Keywords:** chronic hepatitis B, antiviral therapy, immunotherapy, woodchuck, woodchuck hepatitis virus

## Abstract

Infection with hepatitis B virus (HBV) is responsible for the increasing global hepatitis burden, with an estimated 296 million people being carriers and living with the risk of developing chronic liver disease and cancer. While the current treatment options for chronic hepatitis B (CHB), including oral nucleos(t)ide analogs and systemic interferon-alpha, are deemed suboptimal, the path to finding an ultimate cure for this viral disease is rather challenging. The lack of suitable laboratory animal models that support HBV infection and associated liver disease progression is one of the major hurdles in antiviral drug development. For more than four decades, experimental infection of the Eastern woodchuck with woodchuck hepatitis virus has been applied for studying the immunopathogenesis of HBV and developing new antiviral therapeutics against CHB. There are several advantages to this animal model that are beneficial for performing both basic and translational HBV research. Previous review articles have focused on the value of this animal model in regard to HBV replication, pathogenesis, and immune response. In this article, we review studies of drug development and preclinical evaluation of direct-acting antivirals, immunomodulators, therapeutic vaccines, and inhibitors of viral entry, gene expression, and antigen release in the woodchuck model of CHB since 2014 until today and discuss their significance for clinical trials in patients.

## 1. Introduction

Hepatitis B virus (HBV) is a hepatotropic, non-cytolytic DNA virus classified under the genus *Orthohepadnavirus* in the family *Hepadnaviridae* [1,2]. HBV is an enveloped virus with a nucleocapsid containing a 3.2 kilobase (kb) long genome of partially double-stranded (ds) or relaxed circular (rc) DNA [1,3]. HBV enters hepatocytes by an initial low affinity binding of virions to heparin sulfate proteoglycans (HSPGs) located on the cell surface, followed by a high affinity binding of the large HBV surface (HBsAg) protein to the bile acid transporter, sodium taurocholate co-transporting protein (NTCP) [4,5]. In addition to NTCP as an essential receptor, the epidermal growth factor receptor (EGFR) has been identified as a co-receptor for HBV entry into hepatocytes [6]. Following binding and internalization of HBV virions, the genome within the nucleocapsid is transported to the nucleus for completing rc-DNA into covalently closed circular DNA (cccDNA) using the host DNA polymerase II and the cellular DNA repair machinery [7,8]. The cccDNA is the reservoir of the HBV genome in the nucleus and is used for the transcription of all viral genomic and sub-genomic RNAs. The 3.5 kb transcript of pre-genomic RNA (pgRNA) functions as the template for new progeny viral rc-DNA but also serves as a mRNA for the translation of the viral polymerase/reverse transcriptase and the core protein. The HBV precore protein is translated from the precore mRNA, another viral transcript that is slightly longer than the pgRNA, and serves as the precursor of the soluble HBV e antigen (HBeAg) and the recently characterized PreC antigen [9,10]. Furthermore, the large (L-HBsAg), middle (M-HBsAg), and small HBsAg (S-HBsAg) proteins are translated from 2.4 kb and 2.1 kb transcripts, while the HBV x antigen (HBxAg) is translated from a 0.7 kb transcript [7,11]. The pgRNA along with the viral polymerase/reverse transcriptase are packaged into newly formed nucleocapsids consisting of core proteins (HBcAg). Encapsidation initiates the reverse transcription of pgRNA to negative strand DNA followed by the synthesis of positive strand DNA [3]. These mature nucleocapsids containing HBV rc-DNA are either enveloped by HBsAg proteins and secreted as complete virions or are recycled back to the nucleus for replenishment of the cccDNA pool [12,13]. Recent studies have investigated integration events of the HBV genome into host chromosomal DNA and found that such viral DNA serves as an alternate source of HBsAg [14]. The increased oxidative stress present during chronic hepatitis B (CHB) facilitates this integration and, as a secondary reservoir for HBsAg (besides cccDNA), it adds to the challenge of overcoming T-cell exhaustion due to prolonged exposure to viral antigens. More studies are needed for characterizing the properties of HBsAg derived from the integrated HBV DNA and for determining their implication on immunopathogenesis.

The World Health Organization (WHO) has recognized HBV infection as a serious public health concern that is affecting nearly 296 million people worldwide who are chronic carriers of HBV [15]. HBV-related liver disorders, including CHB, fibrosis, cirrhosis, and primary cancer or hepatocellular carcinoma (HCC), account for nearly one million deaths each year thereby surpassing other infectious diseases, such as tuberculosis and acquired immunodeficiency syndrome (AIDS) caused by infections with Mycobacterium tuberculosis or human immunodeficiency virus (HIV), respectively [16]. With the lack of curative treatment interventions, reducing the incidence of HBV infection currently depends on prophylactic vaccination. In the past three decades, vaccine administration programs throughout the world have made great progress to protect the majority of the population against HBV, especially those that are based on a triple-dose regimen of HBsAg-containing vaccines [17]. Current treatment options against CHB are limited to oral nucleos(t)ide analogs (NAs) and subcutaneous (sc), pegylated interferon-alpha (pegIFN-α) [18]. Since NAs do not directly target the cccDNA and only interfere with the function of the HBV polymerase, prolonged or even life-long administration is required to avoid viral relapse after treatment discontinuation. Entecavir (ETV), tenofovir disoproxil fumarate (TDF), and tenofovir alafenamide fumarate (TAF) are currently recommended as first-line drugs for NA treatment against CHB. They are effective in controlling viral replication without the development of drug resistance but the percentage of patients achieving functional cure, which is defined as undetectable serum HBV DNA and HBsAg with or without seroconversion, is <10% even after extended treatment [19]. Contrary, pegIFN-α therapy for one year mediates a sustained off-treatment antiviral response in approximately 10–20% of patients. While this pleiotropic cytokine directly targets the cccDNA and triggers natural killer (NK) cell functions [20], the obstacles involved with its systemic administration include unpredictable treatment outcomes and severe adverse effects resulting in low patient compliance. Many factors, such as age, HBeAg status, HBV genotype, and serum levels of HBV rc-DNA and the liver enzyme alanine transaminase (ALT), influence the individual responsiveness to pegIFN-α treatment [21]. Thus, future antiviral drugs need to address both the safety and efficacy concerns of current anti-HBV therapeutics for designing shorter and more effective treatment options against CHB. Towards this goal, several direct-acting antivirals (DAAs) have been developed to target specific steps of the HBV life cycle, including viral entry, capsid formation, and protein transcription and release [22,23]. However, the failure of these DAAs to directly target the cccDNA sustains the challenge of viral relapse after treatment discontinuation. Since HBV pathogenesis and liver disease progression depend on the host antiviral immunity, as described below in more detail, several approaches to modulate the impaired immune response during CHB have been developed, including agonistic stimulation of innate immune receptors, adoptive T-cell transfer, and therapeutic vaccination. Several drug candidates, consisting of both DAAs and immunomodulators, have shown promise in preclinical studies in animal models of HBV and are currently evaluated in clinical trials in patients with CHB.

**Figure 1:**
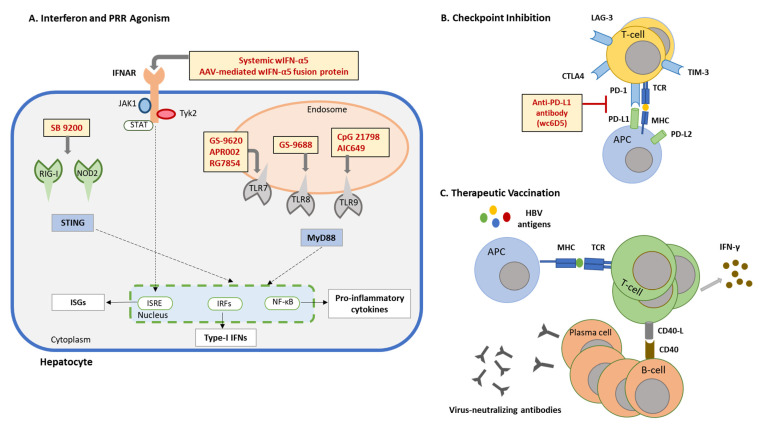
Immunomodulatory strategies recently evaluated in the woodchuck animal model of CHB. Approaches to retrieve the impaired antiviral immune response for the control of chronic HBV infection include (**A**) Interferon and PRR Agonism with IFN-α (systemic wIFN-α5 and AAV-mediated wIFN-α5 fusion protein), RIG-I/NOD2 agonist (SB 9200), TLR7 agonists (GS-9620, APR002, and RG7854), TLR8 agonist (GS-9688), and TLR9 agonists (CpG 21798 and AIC649). These compounds activate their receptor downstream signaling pathways via the adaptor molecules STAT, STING, or MyD88 for the induction of type-I IFNs, ISGs, and pro-inflammatory cytokines. Type-I IFNs target the persistent viral cccDNA reservoir in the nuclei of infected hepatocytes. (**B**) Checkpoint Inhibition with anti-PD-L1 antibody (wc6D5). This leads to the blockage of inhibitory PD-1/PD-L1 interaction between T-cells and APCs. (**C**) Therapeutic Vaccination. Protein- or DNA- based vaccines containing or encoding for viral antigens are administered for the priming/activation of antiviral T- and B-cells. T-cells secrete antiviral cytokines, including IFN-γ, and B-cells differentiate into plasma cells and secrete virus-neutralizing antibodies thereafter. See text for details. Abbreviations: AAV, adeno-associated virus; APC, antigen presenting cell; CD40, B-cell surface antigen; CD40-L, CD40 ligand; CTLA4, cytotoxic T-lymphocyte antigen 4; HBV, hepatitis B virus; IFN, interferon; IFNAR, interferon-alpha receptor; IRFs, interferon regulatory factors; ISGs; interferon-stimulated genes; ISRE, interferon-sensitive response element; JAK1, janus kinase 1; LAG-3, lymphocyte-activation gene 3; MHC, major histocompatibility complex; MyD88, myeloid differentiation primary response 88; NF-κB, nuclear factor kappa B transcription factor; NOD2, nucleotide binding oligomerization domain containing 2; PD-1, programmed cell death protein 1; PD-L1, PD-L2, PD-1 ligands 1 and 2; PRR, pathogen recognition receptor; RIG-I, retinoic acid-inducible gene I; STAT, signal transducer and activator of transcription; Tyk2, tyrosine kinase 2; STING, stimulator of interferon genes; TCR, T-cell receptor; TIM-3, T-cell immunoglobulin and mucin domain-containing protein 3; TLR, toll-like receptor; wIFN-α5, recombinant woodchuck IFN-α5.

**Figure 2:**
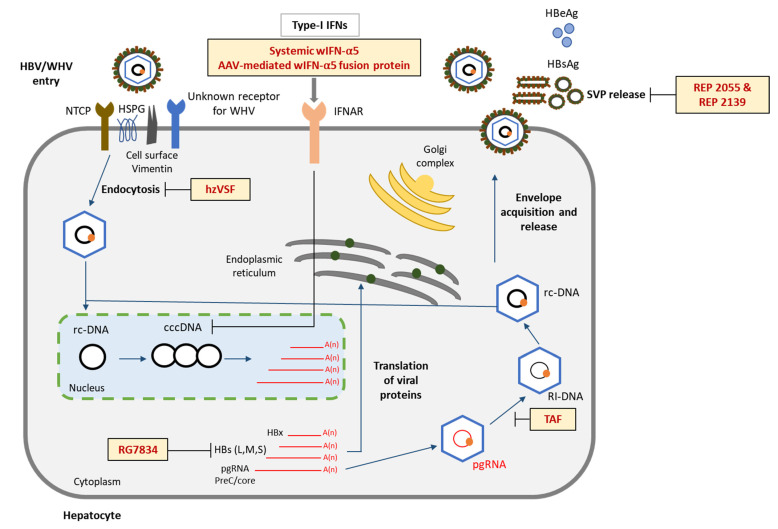
Antiviral drugs targeting different steps of the HBV/WHV life cycle recently tested in the woodchuck animal model of CHB. Attachment to and entry of HBV into hepatocytes is mediated by binding to HSPG and NTCP followed by a multi-step endocytosis process. Viral infection induces the expression of cell surface vimentin, which is known to support endocytosis. The monoclonal antibody hzVSF targeting cell surface vimentin inhibits the internalization and hence the endocytosis of HBV/WHV thereby acting as a viral entry inhibitor. RG7834 is a small molecule inhibitor of HBV gene expression that targets the poly (A) polymerases PAPD5 and PAPD7 to destabilize and degrade viral RNAs, especially surface mRNA. Similar to other NAs, TAF targets the HBV/WHV polymerase and inhibits viral DNA synthesis. REP 2055 and REP 2139 are nucleic acid polymers that target the assembly of HBV subviral particles and block the release of HBsAg from infected hepatocytes. Unlike these DAAs and NAs, type-I IFNs and systemic wIFN-α5 and AAV-mediated wIFN-α5 fusion protein directly target the persistent viral cccDNA reservoir in the nuclei of infected hepatocytes. See text for details. Abbreviations: A(n), viral RNAs containing a poly-adenine tail; AAV, adeno-associated virus; cccDNA, covalently closed circular DNA; HBeAg; HBV e antigen; HBs, HBV surface gene encoding for the large (L), middle (M), and small (S) surface antigens; HBsAg, HBV surface or envelope antigen; HBV, hepatitis B virus; HBx, HBV x gene; HSPG, heparin sulfate proteoglycans; hzVSF, humanized virus suppressing factor or viral entry inhibitor; IFN, interferon; IFNAR, interferon-alpha receptor; NTCP, sodium taurocholate co-transporting protein; pgRNA, pre-genomic RNA; PreC/core, HBV PreC/core genes; rc-DNA, relaxed circular DNA; REP 2055 and REP 2139, inhibitors of HBV subviral particle assembly and surface antigen release; RG7834, inhibitor of HBV gene expression, RI-DNA, replicative intermediate DNA; SVP, subviral particles; TAF, tenofovir alafenamide fumarate; WHV, woodchuck hepatitis virus; wIFN-α5, recombinant woodchuck IFN-α5.

Among the various challenges involved in studying HBV pathogenesis and developing curative therapies against CHB, the lack of a small, immunocompetent, HBV-susceptible laboratory animal model is the most fundamental one. The Eastern woodchuck (*Marmota monax*), naturally or experimentally infected with woodchuck hepatitis virus (WHV), develops a similar immunopathogenesis and disease outcome as observed during HBV infection in humans [24]. For more than four decades, this animal model has been used for understanding HBV biology but has also been extensively applied in preclinical studies for evaluating the safety and therapeutic efficacy of novel anti-HBV drugs. In this article, we review the preclinical studies conducted with new anti-HBV drug candidates in the woodchuck animal model during the past eight years (Figure 1 and Figure 2) and discuss the significance of the derived results towards moving these antivirals into clinical trials and the capacity of the woodchuck to predict the overall treatment outcome in patients with CHB.

## 2. The Woodchuck Animal Model of CHB

The Eastern woodchuck belongs to the genus *Marmota* and is a rodent of the *Sciuridae* family that largely habitats the Northeastern regions of the United States and Canada [25]. Natural and experimental infection of these animals with WHV results in similar liver disease progression as observed during HBV infection, including the development of acute and chronic hepatitis B and primary liver cancer [24,25,26]. The first description of WHV infection in woodchucks was published by Summers and colleagues in 1978 after they observed an increased mortality due to HCC in a colony of woodchucks maintained at the Penn Rose Zoo in Philadelphia [27]. This was followed by many studies to characterize WHV replication and associated liver disease in greater detail in these animals [28,29,30]. Similar to HBV, WHV is classified under the genus *Orthohepadnavirus* [2] because of the similarity in morphological structure, genome organization, and replication mechanism [24]. Furthermore, the outcome of WHV infection in woodchucks is also age-dependent as in HBV-infected humans. While WHV infection in adult woodchucks leads to acute hepatitis B (AHB) and subsequent resolution in more than 95% of animals, infection in neonatal woodchucks usually becomes persistent and results in CHB in 65–75% of animals [28]. Besides transient, self-limited and chronic WHV infections involving the development of AHB or CHB, respectively, the woodchuck is also suitable for studying HBV-induced HCC [30,31]. Application of these animals in the HCC treatment of patients is evolving due to suitable liver size and comparable vasculature, especially for the development of novel anti-cancer drugs and new approaches for tumor-targeted treatment [31].

At the begin of HBV research, experimental infection of chimpanzees was applied as an animal model for studying the host adaptive immune response and for testing the safety and efficacy of vaccines and antiviral therapeutics [32]. However, chimpanzees are an endangered species, and thus are no longer permitted for use in research in many countries due to ethical reasons. Several inbred HBV mouse models, including transgenic, transduced, and humanized chimeric mice, are mainly used now in HBV research; however, mice are not a natural host of HBV, and thus do not support all steps of the viral life cycle, including the formation of cccDNA [33]. The dually humanized mouse models are immunocompetent, but additional studies are needed to confirm the establishment of chronic HBV infection and disease progression to CHB and HCC [33]. Recent work in rhesus macaques demonstrated that the hepatocytic expression of human NTCP enables susceptibility to HBV, but the infection appears to be only transient [34], although it is extendable after depletion of Kupffer cells and repression of inflammation in the liver, and with the prolonged use of immunosuppressants to inhibit antiviral immune response [35]. The restricted access to chimpanzees and macaques, in addition to the narrow range of natural hosts for HBV, has led to the use of alternative animal models, including Pekin ducks, squirrels, and woodchucks. The woodchuck has several advantages over other surrogate animal models. Outbred woodchucks are a natural, immunocompetent host of an HBV-related hepadnavirus and support all steps of viral replication, as observed for HBV. WHV infection in woodchucks can be modeled for studying both acute and chronic outcomes of HBV infection in humans, and WHV-induced liver disease and progression to HCC largely resembles those in humans due to HBV infection. The availability of woodchucks from only a few academic and commercial sources and the lack of many host-specific, protein-based reagents are two limitations that need to be addressed in the future. However, the recent identification of the woodchuck transcriptome [36,37] and genome [38,39] will greatly support the latter effort. Current NA-based antiviral therapies approved for treatment of CHB in patients were all evaluated in woodchucks [24,40], and ETV was solely developed in this animal model [41]. These preclinical studies were critical in determining the therapeutic efficacy and, more importantly, the safety of NAs before evaluation in clinical trials. The studies also demonstrated the capacity of the woodchuck to predict the treatment outcome in patients [42,43]. Similarly, in the current era of immunomodulatory drug development against HBV, immunocompetent animal models, such as the woodchuck, are a prerequisite for delineating the antiviral host innate and adaptive immunity induced or retrieved by treatment with these novel therapeutics.

## 3. Immunopathogenesis of HBV/WHV Infection

HBV pathogenesis is driven by viral determinants and associated host immune responses. Early studies in chimpanzees demonstrated a lack in type-I IFN response and a failure to induce IFN-stimulated genes (ISGs) immediately after HBV infection [44,45]. However, the perception of HBV as a stealth-like virus continues to be a subject of controversy, as more recent studies describe the recognition of HBV by pathogen recognition receptors (PRRs) [46,47,48] and the interference of HBV proteins with the antiviral host innate immunity [49,50]. While these mainly in vitro studies have shown that almost all HBV proteins, such as HBsAg, HBeAg, HBxAg, and HBV polymerase, can suppress the innate immune response by interference with the downstream signaling pathways of PPRs, another study using liver biopsy samples from patients found that HBV neither activates nor actively inhibits these immune processes [51]. This feature of HBV, as well as its non-cytopathic nature, then leads to an asymptomatic phase in the early weeks of infection. This is followed by increased HBV replication and AHB development in adults resulting in immunological control of the infection, and viral resolution usually does not cause any serious symptoms, such as acute liver failure [52]. Contrary, CHB, which is observed in HBV infections that were acquired by vertical transfer from infected mother to baby or early in life as an infant, is mainly a consequence of prolonged exposure to viral proteins that results in a failure to mount an effective antiviral immune response [53,54,55,56]. WHV infection in woodchucks presents with similar pathogenesis and host immune response as observed in human HBV infections. Acute, self-limited WHV infection in adult woodchucks also shows an initial lag phase in viral replication followed by a transient increase in viral load, with maximum titers of serum viremia and antigenemia before the establishment of AHB and subsequent viral resolution [9,57,58,59]. Recent studies describing the kinetics of WHV markers in serum and liver and the associated innate and adaptive immune responses during AHB and resolution revealed that the initial reduction in WHV replication correlates with an increased expression of NK-cell markers and IFN-γ [60,61]. Following this, the marked decline in peripheral and intrahepatic WHV markers was associated with maximum elevations in liver enzymes, increased expression of IFN-γ, cytolytic effector molecules, markers of immune cell subsets, including NK-cells, macrophages/Kupffer cells, T-helper (T_H_) cells, cytotoxic T-cells (CTLs), and B-cells, and seroconversion to antibodies against WHV surface (WHsAg) and WHV e (WHeAg) antigens (anti-WHs and anti-WHe antibodies). Similar to patients with CHB, the functions of immune cells are impaired in woodchucks with established chronic WHV infection. In direct comparison to the peak expression of various immune cell markers during AHB in woodchucks, their expression during CHB is significantly reduced [57,60]. Comparable to chronic HBV infection, which has been extensively characterized by weak, virus-specific CD4+ and CD8+ T-cell responses, both qualitatively and quantitatively [62,63,64], previous studies in woodchucks also demonstrated the lack of such T-cell responsiveness during CHB [65,66]. Seroconversion to antibodies against viral antigens is considered the hallmark for successful immunological control of HBV and WHV infection, but virus-neutralizing antibodies to the viral surface proteins are either absent or only weakly present in patients and woodchucks with CHB [28,67]. While there is a better understanding of the role of adaptive immune response during HBV infection, especially the antiviral functions of T-cells, recent development and therapeutic efficacy of compounds targeting PRRs in preclinical animal studies have created an interest in exploring the role of innate immunity in greater detail. As mentioned above, whether or not HBV is recognized by PRRs remains controversial, and the mechanisms involved in this process are less understood. Studies have described the activation of toll-like receptors (TLRs), such as TLR2 and TLR4, by HBV proteins, including HBsAg and HBcAg-containing nucleocapsids empty of HBV rc-DNA [68,69]. Furthermore, cytosolic PRRs, including retinoic acid-inducible gene I (RIG-I) and cyclic GMP-AMP synthase (cGAS), can recognize the epsilon structure of the HBV pgRNA or high concentrations of HBV DNA, respectively [48,70]. In woodchucks, activation of an innate immune response in the liver was observed within a few hours after experimental WHV infection, leading to a partial inhibition of viral replication but not to immunological control, and the infection subsequently expanded further [58]. In a more recent study, the kinetics of many PRRs during WHV resolution in woodchucks were characterized and their peak intrahepatic expression and presence compared to those in WHV-naïve and WHV-infected animals with CHB [71]. In line with the stealth-like behavior of HBV, no significant upregulation in PRR transcription and translation were observed five weeks after experimental WHV infection, but a maximum was noted during 9–13 weeks post-infection, at the time of AHB and subsequent WHV resolution. In addition, the expression of type-I IFNs, ISGs, and several innate immune cell subsets was unchanged at five weeks post-infection but markedly increased thereafter. Most PRRs and other innate immune response markers were significantly reduced in woodchucks with CHB.

## 4. Nucleos(t)ide Analog Treatment in Woodchucks

Previous studies in the woodchuck have evaluated the safety and therapeutic efficacy of NAs and combinations thereof [24,40]. More recently, monotherapy with TAF (Vemlidy) was tested for the first time in this animal model [72] (Figure 2, Table 1). Like TDF, TAF is also a prodrug of tenofovir, but safer and antiviral efficacious at a much lower dose in patients with CHB [73]. In the woodchuck study, animals with CHB were treated daily with oral TAF for twelve weeks. TAF treatment at a high dose resulted in marked reductions of serum WHV DNA, WHsAg, and WHeAg at the end of treatment that were significant when compared to the pretreatment baseline. In addition, TAF treatment reduced viral nucleic acids in the liver, and the declines in WHV DNA replicative intermediates (RI-DNA) and WHV cccDNA, but not in WHV surface and pg RNAs, were also significant. Intrahepatic presence of cytoplasmic WHV core antigen (WHcAg), but not of membranous WHsAg, was further significantly reduced at the end of treatment. The pronounced antiviral effect, however, was only transient, as a rebound in serum and liver viremia and antigenemia was observed immediately after drug withdrawal. Comparable to previous NA applications in woodchucks with CHB [24], TAF treatment did not lead to seroconversion, as anti-WHs and anti-WHe antibodies were undetectable throughout the study. Regarding disease progression, transient declines in liver inflammation (i.e., portal and sinusoidal hepatitis) were noted during treatment. TAF administration was well-tolerated at the applied dose, and changes in body weight, body temperature, and hematology and clinical chemistry parameters, including the liver enzymes ALT and aspartate aminotransferase (AST), were absent. Another liver enzyme, sorbitol dehydrogenase (SDH), however, declined significantly during treatment, indicating together with the reduced liver inflammation that the progression of CHB was temporarily slowed down by TAF. Many preclinical studies in woodchucks have used NAs for either modeling the treatment outcome in patients or, more recently, for reducing viremia before or during parallel administration of novel experimental drugs. The additional therapeutic benefit associated with combining NAs and DAAs or immunomodulators is described below.

## 5. Immunomodulation in Woodchucks

The current treatment options for CHB involving NAs are safe and antiviral effective but require most likely infinite administration in most patients as they are rarely curative. Immunotherapy on the other hand provides the unique opportunity to address this limitation, because it has the potential of directly targeting the cccDNA and controlling the viral infection by the induced host immune response. The rather diverse approaches for modulating both the innate and adaptive arms of antiviral immunity have been reviewed previously in detail [76,77]. In this section, recent preclinical studies on immunomodulation in the woodchuck are discussed, including treatment with IFN-α, small agonist molecules targeting selected PRRs, immune checkpoint inhibitor, and therapeutic vaccine (Figure 1 and Figure 2, Table 2).

### 5.1. IFN-α Therapy

Systemic IFN-α administration: The groundwork for immunomodulation against CHB in patients started with systemic IFN-α therapy. This multi-functional cytokine is secreted by many immune cell subsets and mediates a direct antiviral effect against various viral infections, including HBV, and can additionally induce a broad, antiviral immune response via the upregulation of ISGs [89]. Importantly, IFN-α has been shown to inhibit HBV replication by epigenetic repression of cccDNA transcription [90]. While IFN-α therapy can be a single and finite treatment option for some patients, there are several obstacles that need to be addressed, including HBV genotype-specific treatment outcome, liver disease status, and possible adverse effects before using it as a personalized medicine for CHB [21]. Following the identification of the blood and liver transcriptome and the initial characterization of the innate and adaptive immune responses involved in AHB and resolution of WHV infection in woodchucks [36,37,91], another study by Fletcher and colleagues reported the intrahepatic gene signatures associated with a successful treatment response to systemic IFN-α administration in these animals [78]. After a dose-finding study in WHV-naïve woodchucks, recombinant woodchuck IFN-α5 (wIFN-α) was administered sc three times per week for 15 weeks to woodchucks with CHB. The first seven weeks of treatment included a low wIFN-α dose. Since an interim analysis did not reveal an immediate, marked antiviral effect, the original dose was increased by five-fold and administered for the remaining eight weeks of treatment. Compared to the low dose, the high dose of wIFN-α induced a rapid antiviral effect, with significant declines in serum viremia and antigenemia in many woodchucks. Correlating with these declines in serum viral markers, wIFN-α treatment also reduced all viral nucleic acids in the liver of these animals, including WHV RI-DNA, cccDNA, and surface and pg RNAs. However, the antiviral effect mediated by wIFN-α was only transient and WHV relapsed after treatment discontinuation in most woodchucks although at varying degrees. Consistently elicited anti-WHs antibodies were only detected in the serum of one animal with a more pronounced, and importantly, sustained antiviral response (SVR) post-treatment. Tolerability issues or adverse effects were not noted; however, increases in the liver enzymes ALT and AST were present in individual woodchucks, especially during treatment with the high wIFN-α dose. While these fluctuations in transaminases did not correlate well with the peak antiviral response, a temporal association with increased liver inflammation was observed for many animals. A dose-dependent elevation in blood ISG expression was noted, but only the high wIFN-α dose induced the transcription of various T helper cell type 1 (T_H_1) cytokines in the periphery. Among all cytokines induced, the magnitude of IFN-γ expression appeared to be associated with response, partial response, and no response of woodchucks to wIFN-α treatment. The intrahepatic transcriptional profiles, as determined by RNA-sequencing (RNA-seq), revealed that wIFN-α treatment mediated expression changes in genes associated with IFN response and immune cell subsets migrating into and/or proliferating within the liver. Interestingly, these gene signatures also indicated that the antiviral response was less modulated by ISGs, but selectively more so by the response of NK- and CTLs in the liver. It further appeared that both non-cytolytic and cytolytic functions of intrahepatic NK- and T-cells were improved in responder woodchucks, and thus involved in the successful suppression of WHV by wIFN-α. Altogether, this study found important parallels between the IFN-α treatment response of woodchucks and patients with CHB and established the translational value of the woodchuck animal model in characterizing the immune correlates of treatment with this cytokine. The study further provided rationale for evaluating other immunomodulators, including agonists of various PRRs, for the induction of endogenous type-I IFNs.

Adeno-associated virus-mediated IFN-α administration: The severe adverse effects frequently observed during systemic IFN-α administration in patients are the major challenge in using this cytokine as a treatment option against CHB. For testing if the peripheral cytokine exposure and associated immunotoxicity after sc injection can be limited, liver-targeted transduction of IFN-α was performed in mice and woodchucks with an adeno-associated virus (AAV) vector [79]. Since a previous mouse study has shown that the fusion of IFN-α to apolipoprotein A-I maintained the antiviral and immunomodulatory functions of the cytokine without causing toxicity related to hematology and the central nervous system [92], the safety and feasibility of liver-specific delivery of this fusion protein was tested. Viral vectors containing IFN-α, without (i.e., AAV-IFN) or with fusion to apolipoprotein A-I (i.e., AAV-IA) were intravenously (iv) administered once to wildtype mice. The study revealed that a single dose of AAV-IA induced higher serum IFN-α levels than AAV-IFN and was without severe peripheral toxicity. This was in contrast to a single dose of AAV-IFN, as all mice died within 60 days after the administration and mortality was associated with significant declines in erythrocytes, leukocytes, platelets, neutrophils, and monocytes. In separate mouse studies, it was further found that both vectors induced similar levels of ISGs in the liver, such as interferon-stimulated gene 15 (ISG15) and 2’-5’-oligoadenylate synthetase 1 (OAS1), and equally protected wildtype mice against a lethal challenge with encephalomyocarditis virus; however, iv AAV-IA administration was again associated with less hematological toxicity and absent histological changes in the liver. In HBV transgenic mice, a single iv injection of these vectors induced IFN-α in serum at levels that were higher with AAV-IA than AAV-IFN, but both vectors significantly reduced serum viremia and antigenemia at a comparable magnitude. Both vectors also significantly and similarly reduced intrahepatic HBV RI-DNA and HBcAg RNA in the liver; however, AAV-IFN administration again was associated with leukopenia, anemia, and thrombocytopenia. In woodchucks with CHB, the same AAV vectors encoding wIFN-α5, alone (AAV-wIFN) or fused to woodchuck apolipoprotein A-I (AAV-wIA), were administered once by intrahepatic injection at two separate doses. While the dose-dependent elevation in serum IFN-α was comparable between both vectors, all animals treated with AAV-wIFN needed to be euthanized due to severe pancytopenia. Among the woodchucks administered AAV-wIA, one animal was euthanized because of HCC development, but treatment in the other animals was without marked changes in hematology and liver enzymes. A pronounced antiviral effect on WHV replication was not mediated by either vector, as the high pretreatment loads of serum WHV DNA only declined moderately. While these results validated the use of AAV vectors for treatment of CHB and demonstrated a superior safety of AAV-IA over AAV-IFN, they also suggested that reducing serum WHV DNA before the vector administration may be beneficial, as a high viremia level is associated with a diminished response to IFN-α therapy in patients [93]. Thus, a subsequent woodchuck study tested the antiviral effect of AAV-wIA in the setting of low viremia. Woodchucks with CHB received a single iv injection of either AAV-wIA or a control vector encoding woodchuck apolipoprotein A-I (AAV-wApo), together with oral ETV administered daily for four weeks. As expected, ETV treatment in both groups resulted in marked but comparable declines in serum WHV DNA, while the reductions in serum WHsAg were only modest. Following ETV withdrawal, viremia and antigenemia immediately rebounded in all surviving woodchucks treated with AAV-wApo. This was in contrast to woodchucks administered AAV-wIA in which WHV relapse was significantly delayed for several weeks, with two animals that had still suppressed WHV DNA at the end of the study. Seroconversion to anti-WHs antibodies, however, did not occur in any animal. AAV-wIA treatment of woodchucks was safe and well-tolerated, and without detectable IFN-α in serum and aberrant changes in hematology and liver enzymes. Thus, prolonged, AAV-mediated liver expression of an IFN-α fusion protein in combination with NA treatment may be considered as an alternative to systemic IFN-α administration for the treatment of CHB. Although comparable vectors have been applied in clinical trials for different diseases [94], AVV-mediated therapy with IFN-α fused to apolipoprotein A-I has not been evaluated in patients with CHB so far.

### 5.2. TLR7 Agonists

TLRs are the earliest discovered, and thus the most widely studied family of PRRs. Human TLR7 is a transmembrane receptor in the endosomal compartment of cells that recognizes viral single-stranded (ss) RNA and then triggers the downstream signaling pathway to induce a type-I IFN response, especially in plasmacytoid (p) dendritic cells (DCs) and B-lymphocytes [95]. Production of IFN-α, T-cell-attractant chemokines, such as IFN-γ-induced protein 10 (IP-10) or C-X-C motif ligand 10 (CXCL10), and often pro-inflammatory cytokines subsequently results in the activation of NK-cells and cross-priming of CTLs leading to the induction of both antiviral innate and adaptive immune responses [96,97].

GS-9620: In regard to anti-HBV drug development, GS-9620 was the first-in-class TLR7 agonist developed by Gilead Sciences for modulating the impaired host immune response in patients with CHB [98]. This orally active, synthetic, small agonist molecule was initially tested in chimpanzees. In the study by Lanford and colleagues, oral administration of GS-9620 three times per week for four weeks, and then again for another four weeks with a two-fold higher dose after a rest period of one week, mediated an antiviral effect in the serum of all chimpanzees with chronic HBV infection, and in the liver of at least one animal [99]. The reductions in serum viremia and additionally in HBsAg and HBeAg, as well as the induction of an antiviral immune response in the liver, including type-I IFNs, ISGs, NK-cells, and T-cell subsets, overall led to a SVR in most animals for up to four months, but was not accompanied by seroconversion to anti-HBs antibodies. Thereafter, the pharmacokinetics (PK), pharmacodynamics (PD), therapeutic efficacy, and safety of GS-9620 were evaluated in the woodchuck [80]. In an initial dose-finding study, groups of WHV-naïve woodchucks received orally a single, accelerating GS-9620 dose. The largely dose-dependent distribution of GS-9620 in the periphery resulted in an increased expression of OAS1 and IFN-induced guanosine-5’-triphosphate (GTP) binding protein (MX1) in blood, indicating IFN-α induction by the TLR7 agonist. In the subsequent efficacy study, woodchucks with CHB orally received two separate GS-9620 doses for a total of four or eight weeks, depending on the dosing frequency (i.e., every other day, every other day in every other week, or once a week). In some groups, treatment was halted after the initial two weeks of dosing due to elevations in liver enzymes and thrombocytopenia that reversed during a treatment interruption of nine to ten days, and treatment was reinitiated thereafter with half of the original dose. GS-9620 administration significantly increased the transcription of OAS1 and MX1 in the blood of most woodchucks, indicating again activation of TLR7 by the agonist. In the group with the most pronounced antiviral effect (i.e., Group 3), GS-9620 treatment rapidly and markedly reduced serum WHV DNA loads that were significant when compared to the pretreatment baseline. The reductions in serum viremia were accompanied by a loss of detectable WHsAg in all woodchucks and significant declines in intrahepatic WHV DNA and RNA molecules. At the end of the study in week 31, all woodchucks of Group 3 demonstrated an SVR, and a subset of animals seroconverted to anti-WHs antibodies. Compared to placebo-treated control woodchucks, GS-9620 treatment further significantly reduced the incidence of HCC development in animals with an SVR. Tolerability issues or adverse effects other than reversible thrombocytopenia were absent, but liver enzymes were transiently elevated during treatment in most woodchucks and coincided with the reductions in WHV markers, especially of intrahepatic WHV cccDNA. RNA-seq of liver tissues from woodchucks with an SVR revealed the activation of an antiviral immune response that was based on the induction of type-I and type-II IFNs and of NK-cells, CD8+ T-cells, and B-cells. Together with the elevation in transaminases, this suggested that the GS-9620-induced antiviral effect was likely mediated by the non-cytolytic and cytolytic activity of intrahepatic NK- and T-cells, as well as the activation of B-cells. While the studies in chimpanzees and woodchucks demonstrated promising therapeutic efficacy against chronic HBV infection, and thus justified further evaluation of the TLR7 agonist in clinical trials, GS-9620 administration to NA-naïve or -experienced patients with CHB did not mediate an antiviral effect at the applied dose range [100,101,102], while higher doses were associated with severe tolerability issues [101]. The apparent discrepancy in the GS-9620-mediated treatment outcome in chimpanzees and patients is currently unknown; however, a follow-up study in woodchucks [83] discovered that the unprecedented antiviral effect achieved in this animal model with any single agent tested so far was due to an additional activation of TLR8 by high GS-9620 dosage. The clinical trials and the overall treatment outcome with GS-9620 and other novel anti-HBV compounds in patients are discussed below in more detail.

APR002: For addressing the limitations of using sub-therapeutic agonist doses due to off-target effects by unintended TLR7 activation in the periphery, the liver-targeting TLR7 agonist, APR002, was designed by Apros Therapeutics [81]. This compound contains a liver-specific moiety for enhanced hepatic uptake and retention and reduced systemic exposure in the blood. A single, oral or iv dose administration of the TLR7 agonist to wildtype mice confirmed this PK profile of APR002 that is distinct to that of GS-9620. APR002 also induced lower levels of pro-inflammatory cytokines in mice following a single oral dose administration over a 1000-fold range, including interleukin (IL) 6 and tumor necrosis factor-alpha (TNF-α), that are thought to have contributed to the limited GS-9620 tolerability in patients. Oral administration of a single, increasing dose of APR002 to WHV-naive woodchucks demonstrated a dose-dependent increase of the TLR7 agonist in serum and liver, in addition to the induction of IFN-α and MX1 expression that appeared dose-independent. In the subsequent efficacy study in woodchucks with CHB, the antiviral effect of APR002, alone and together with ETV, was assessed. Woodchucks undergoing oral ETV monotherapy were treated daily for 20 weeks. Animals receiving APR002 monotherapy were treated orally every week for twelve weeks, initially at an intermediate dose for six weeks and then at a two-fold higher dose for another six weeks. Woodchucks administered combination therapy were treated daily with oral ETV for 20 weeks. Starting in week five, animals also received oral APR002 every week for twelve weeks. In one group, APR002 was initially administered at a low dose for seven weeks that was increased by four-fold to the high dose for the remaining five weeks, while another group received APR002 always at the intermediate dose. Monotherapy with APR002 mediated only modest declines in serum viremia and antigenemia. As expected, ETV monotherapy induced a marked effect on serum viremia but also on antigenemia at the selected dose and treatment duration. Combination treatment with ETV and APR002 resulted in reduced serum viremia and antigenemia that was largely comparable to ETV monotherapy. The declines in serum WHV markers achieved by the various treatment regimens were significant when compared to the pretreatment baseline, at least at one to most time points during and after treatment. Following drug withdrawal, WHV replication relapsed in all woodchucks administered ETV or APR002 monotherapy, while an SVR occurred in a subset of animals treated with the ETV/APR002 combination that lasted for at least 16 weeks. Woodchucks with undetectable viremia and antigenemia in serum also demonstrated undetectable or markedly suppressed WHV DNA and RNA molecules in the liver, while anti-WHs and anti-WHe antibodies emerged during and after combination treatment. In contrast to ETV monotherapy, woodchucks treated with APR002, alone and together with ETV, had a marked expression of ISG15 and OAS1 in blood and liver that declined after treatment cessation. Although individual variation was noted in regard to the magnitude of ISG induction, the on-target activation of TLR7 was only evident in animals that received APR002. In addition, woodchucks with an SVR had sometimes marked but transient increases in liver enzymes during add-on APR002 treatment that coincided with the induced antiviral innate and adaptive immune responses. APR002, alone and together with ETV, was safe, although TLR7 agonism was associated with slightly lower body temperatures that normalized after the end of treatment. Overall, the woodchuck study with APR002 was critical in demonstrating the antiviral potential of a liver-targeting TLR7 agonist for the first time, while the combination with ETV indicated that immunological control of CHB can be achieved by parallel interventions targeting different steps in the viral life cycle. APR002 has not been clinically tested in patients with CHB so far.

RG7854: This compound is a double prodrug of a TLR7 agonist developed by F. Hoffmann-La Roche for improving oral bioavailability and limiting inadvertent receptor activation in the gastrointestinal tract [103]. In an initial dose finding study in woodchucks with CHB, animals received orally two separate doses of RG7854 every other day [82]. Since no marked changes in serum viremia and antigenemia were observed immediately after treatment initiation, the low RG7854 dose was increased by four-fold to the high dose starting at week ten. One to six weeks after the dose switch, half of the surviving woodchucks experienced marked declines in serum WHV DNA, WHsAg, and WHeAg and achieved undetectable levels during the 14 weeks of treatment or shortly after drug withdrawal that lasted at least for 11 weeks. These animals also seroconverted to anti-WHs antibodies but anti-WHe antibodies were not detectable. In woodchucks treated with the intermediate RG7854 dose for 24 weeks, a subset of animals demonstrated an antiviral effect starting six to ten weeks after treatment initiation, with complete suppression of serum viremia and antigenemia and seroconversion to anti-WHs and anti-WHe antibodies in one animal, and a transient decline in these serum WHV markers and a transient presence of anti-WHs but not of anti-WHe antibodies in the second animal. Altogether, RG7854 monotherapy resulted in largely dose-dependent, marked declines in serum WHV DNA, WHsAg, and WHeAg. The reductions in serum viremia and WHs antigenemia achieved with the intermediate and high doses were significant when compared to the pretreatment baseline at one to several time points during and after treatment. For evaluating the antiviral benefit associated with the application of the TLR7 agonist as an add-on to NA treatment, woodchucks with CHB received orally the high RG7854 dose every other day together with oral ETV administered daily for 14 weeks. Most animals had rapid and marked declines in serum viremia and antigenemia within one to five weeks after the initiation of combination treatment that were comparable to those noted during RG7854 monotherapy with the same high dose. A subset of animals also achieved long-lasting suppression of serum WHV DNA and loss of WHsAg and WHeAg, along with the emergence of antibodies against both viral antigens that persisted for at least 18 weeks. Seroconversion to anti-WHs antibodies was associated with remarkably high antibody titers. Overall, combination treatment with RG7854 and ETV markedly reduced serum WHV markers and the declines were significant when compared to the pretreatment baseline at several to almost all time points during and after treatment. For a further delineation of the antiviral effect induced by RG7854, alone and together with ETV, in regard to durability, a SVR was defined as serum loads of WHV DNA < 10^3^ genomic equivalents or copy numbers/mL and WHsAg < 5 ng/mL, and with anti-WHs antibodies present at the end of the study. This rather stringent definition revealed that an equal number of woodchucks each in the mono and combination treatment groups experienced a functional cure but also indicated that the inclusion of ETV did not provide a benefit to RG7854-mediated immunomodulation. In woodchucks that received RG7854 and ETV, a pronounced decline in intrahepatic WHV nucleic acids occurred during combination treatment, and WHV DNA and RNA molecules stayed undetectable in the liver of animals with an SVR until the end of the study. Combination treatment was further associated with marked but variable increases in peripheral ISG transcription, including ISG15, OAS, MX1, and CXCL10, following the first dose of RG7854, suggesting on-target activation of TLR7. Furthermore, the proliferative response of T-cells to stimulation with peptides covering the WHcAg or WHsAg was more pronounced, longer-lasting, and detectable in more animals during RG7854/ETV combination than RG7854 monotreatment, indicating an apparent beneficial effect of ETV, as also noted for the higher titers of anti-WHs antibodies produced by plasma cells. TLR7 agonism with RG7854 was safe and well-tolerated by woodchucks based on unchanged body weights and body temperatures; however, the liver enzymes ALT, AST, and SDH transiently and sometimes markedly increased, especially in animals with an SVR induced by RG7854/ETV combination treatment. The transaminase elevations temporally correlated with a transient elevation in liver inflammation. Moreover, dose-dependent neutropenia and thrombocytopenia were noted that reversed after the end of mono and combination treatment. The strong and long-lasting response of WHV-specific B- and T-cells also suggested that the SVR mediated by RG7854 may be different to GS-9620 and APR002 that target gut-associated lymphoid tissues and/or liver following oral administration and intestinal absorption for activating resident pDCs. As established in a mouse model infected with an HBV-encoding AVV, RG7854 mainly targets secondary lymphoid organs after oral administration and activates pDCs, resulting in high numbers of HBV-specific B- and T-cells in spleen and lymph nodes, but is also detected in the liver [104,105]. Based on the safety and therapeutic efficacy observed in the mouse and woodchuck animal models of HBV, RG7854 was tested in healthy Chinese volunteers [103]. Single and multiple oral administrations were safe and resulted in the peripheral induction of ISGs that were comparable to those obtained in woodchucks. These in vitro data established a translational relevance of the woodchuck for evaluating agonists targeting exclusively human TLR7. RG7854 is currently tested in patients with CHB during a phase II clinical trial (NCT04225715) as an add-on to NA treatment, in combination with a capsid assembly modulator or small interfering (si) RNA.

In addition to the above three TLR7 agonists tested in the woodchuck, another TLR7 agonist developed by Janssen Pharmaceuticals was evaluated for safety and therapeutic efficacy in the AAV/HBV mouse model [106]. Due to the encouraging results achieved in mice, the TLR7 agonist was administered to heathy Chinese volunteers for evaluating its safety, tolerability, and PK/PD profiles in a phase Ia clinical trial (CTR20182248 and NCT03285620) [107]. Administration of single, ascending JNJ-4964 doses, sometimes combined with high-fat diet, was safe, tolerated, and induced largely dose-dependent increases in serum ISGs, such as ISG15, MX1, and OAS1. JNJ-4964 has not been clinically tested in patients with CHB so far.

### 5.3. TLR8 Agonist

Similar to TLR7, human TLR8 is also a transmembrane receptor located in the endosome of cells that recognizes viral ss RNA. Unlike TLR7, TLR8 is predominantly expressed in conventional (c) DCs, monocytes, macrophages, neutrophils, and regulatory T cells (T_regs_) [108]. Activation of the TLR8 downstream signaling pathway produces a large array of pro- and anti-inflammatory cytokines, including IFN-γ and TNF-α, that promote direct antiviral effects [109]. The receptor stimulation can also trigger an adaptive immune response via the cytokines IL-12 and IL-18, and an indirect induction of IFN-γ secretion by NK-cells and mucosal associated invariant T-cells (MAITs) [110]. The TLR8 agonist GS-9688 was developed by Gilead Sciences as an oral immunomodulator of the impaired immune response in patients with CHB [111]. In vitro studies using peripheral blood mononuclear cells (PBMCs) from WHV-naïve woodchucks demonstrated an activation of TLR8 by GS-9688 that was comparable to human PBMCs in regard to the induction of various cytokines and ISGs, such as ISG15 and MX1 [83]. These data indicated again the translational relevance of the woodchuck for assessing agonists of human TLRs, including TLR8. Based on a dose-finding study in WHV-uninfected woodchucks, oral GS-9688 at a selected range was chosen as an optimal dose for TLR8 activation [83]. The dose range was determined by the high expression level of macrophage receptor with collagenous structure (MARCO) in blood of WHV-naïve woodchucks and the strong induction of IL-12p40 in serum of these animals 24 or four to eight hours after a single dose administration of GS-9688, respectively. Different to TLR7 agonism with GS-9620 in woodchucks, type-I IFNs were undetectable in serum following TLR8 stimulation. In the subsequent efficacy study in woodchucks with CHB, groups of animals were treated weekly with vehicle or oral GS-9688 at two separate doses for eight weeks. Compared to the low dose, treatment with the high GS-9688 dose resulted in significant and marked reductions in serum viremia and antigenemia from the pretreatment baseline, and WHsAg became undetectable in half of the animals. These woodchucks were characterized as responders with an SVR after drug withdrawal that lasted at least for 24 weeks, while another animal was considered a partial responder with transient WHV marker reduction during treatment, and two other animals were considered non-responders to treatment. Comparable effects were observed for WHV DNA and RNA molecules in the liver, especially for viral cccDNA that was below the detection level in animals with an SVR. All responder woodchucks also elicited anti-WHs antibodies and had a strong proliferation of T-cells in response to stimulation with WHV antigen-specific peptides. The reductions in serum WHsAg in responder and partial responder woodchucks correlated with a transient increase in the liver enzymes SDH and AST and in liver inflammation, which all reversed after the end of treatment. This overall suggested that the cytolytic functions of intrahepatic T-cells were improved during GS-9688 treatment, and thus involved in the SVR, in addition to anti-WHs antibodies secreted by plasma cells. Treatment with the high GS-9688 dose was safe in woodchucks, as marked changes in body weight, body temperature, and hematology and clinical chemistry parameter were absent. However, one animal with an SVR experienced thrombocytopenia during treatment, and GS-9688 dosing was halted for one week but continued with the same dose after platelet counts normalized.

For further delineating the relationship of GS-9688 PK and PD parameters with intrahepatic immunity and treatment response, a second efficacy study was performed in woodchucks with CHB. Groups of animals were treated weekly with vehicle or oral GS-9688 at the high dose for twelve weeks [83]. The marked reductions in serum WHV DNA and WHsAg and the sometimes associated, transient elevation in liver enzymes during TLR8 agonism with GS-9688 were confirmed in a few responder woodchucks with an SVR after treatment cessation that lasted at least for ten weeks. The increase in serum IL-12p40 levels in these animals, however, did not corelate well with the induced antiviral effect. A comparative analysis for identifying differences between responder and non-responder woodchucks indicated that selected intrahepatic gene signatures present at baseline can influence the treatment response to this TLR8 agonist. Especially the basal expression of follicular helper T-cell (T_FH_) markers and the associated cytokine, IL-21, was higher in responder than in non-responder woodchucks to GS-9688 treatment. Altogether, this study demonstrated the possibility of inducing a functional cure by TLR8 agonism with a single agent and further delineated the underlying mechanism of action of GS-9688, including augmentation of a T_FH_ cell response and activation of Kupffer cells in the liver. The study further suggested that the induction of pro- and anti-inflammatory cytokines by TLR8 agonism *versus* type-I IFNs by agonistic TLR7 stimulation has several advantages, including an activation of NK-cells, macrophages, and T-cells in liver without the adverse effects that are common for IFN-α. A subsequent study with PBMCs from healthy volunteers and patients with CHB further dissected the mechanism of TLR8 agonism with GS-9688. The in vitro study confirmed the activation of NK, MAIT, and T_FH_ cells by cytokines secreted after TLR8 stimulation in woodchucks [111]. Thus, the activation of these immune cell subsets, in addition to an improvement of surface antigen-specific B-cells [112], appeared to be a requirement for mediating a sustained reduction in WHsAg and subsequent functional cure by GS-9688 in this animal model. The promising results on PD, safety, and therapeutic efficacy of GS-9688 in the woodchuck justified further evaluation of the TLR8 agonist in patients with CHB during a phase II clinical trial (see below).

### 5.4. TLR9 Agonists

TLR9 is also a transmembrane receptor in the endosomal compartment of cells and recognizes unmethylated cytosine-phosphate-guanine (CpG) motifs in bacterial and viral DNA [113]. The receptor is constitutively expressed in pDCs and B-lymphocytes, but its expression can be induced in additional immune and non-immune cells [114]. Besides the induction of type-I IFNs and the differentiation of B-cells into antibody-secreting plasma cells, TLR9 activation induces secondary effects, such as the secretion of chemokines, activation of NK-cells, and expansion of T-cell subsets [115]. These properties of the receptor downstream signaling pathway make TLR9 a suitable target for immunomodulation.

CpG ODNs: Different classes of CpG oligodeoxynucleotides (ODNs) are widely used agonists of TLR9, as they induce type-I IFNs in pDCs and mediate the proliferation and differentiation of B-cells at varying degrees [116]. In the study by Meng and colleagues, several CpG ODNs developed by Pfizer were tested in vitro for their capability to induce IFN production in woodchuck PBMCs, and the class P ODN, CpG 21798, was selected for further in vivo evaluation [84]. Weekly administrations of sc CpG 21798 to woodchucks with CHB at increasing doses for twelve weeks resulted in a modest, transient reduction in serum viremia that was mainly observed in animals treated with the higher doses of the TLR9 agonist. The associated type-I IFN induction in these animals was somewhat dose-dependent but transient. The study further explored if parallel administration of CpG 21798 and ETV mediates an additional antiviral benefit. Thus, other woodchucks received weekly administrations of sc CpG 21798 for 16 weeks at a high dose together with oral ETV provided daily for twelve weeks. Additional woodchucks were treated with CpG 21798 and ETV alone. Compared to ODN or NA monotreatment, CpG 21798/ETV combination treatment induced faster declines in serum WHV DNA and WHsAg, with undetectable levels in most woodchucks, and viral relapse was delayed by several weeks after treatment discontinuation. Seroconversion to anti-WHs antibodies, however, was absent in all animals. In most woodchucks that received CpG 21798, alone and together with ETV, increased serum levels of IFN were observed during treatment. Correlating with the IFN induction in the periphery, PBMCs of these animals also had durably increased ISG expression, such as MX1, while the transcription of IFN-β and IL-6 was more variable. Most woodchucks treated with CpG 21798, alone and in combination with ETV, presented with unchanged liver enzymes but some animals experienced pronounced elevations in ALT and AST that usually reversed during or after the end of treatment. Since the transaminase increase was not associated with a reduction in serum WHV markers in one woodchuck undergoing CpG 21798 monotherapy, the contribution of these hepatic flares to the outcome of CpG 21798/ETV combination treatment in regard to liver inflammation and/or cytolytic effects by intrahepatic NK- and T-cells is unknown. Overall, CpG 21798 administration in the setting of low viremia mediated by ETV treatment enhanced the antiviral effect of the TLR9 agonist in woodchucks. CpG 21798 has not been clinically tested in patients with CHB so far.

AIC649: This compound is an inactivated parapoxvirus ovis particle preparation developed by AiCuris for the treatment of CHB. Parapoxvirus ovis consists of a dsDNA genome which is rich in cytosines and guanines and thus triggers mainly TLR9 in murine pDCs following complement-mediated opsonization and cellular uptake of viral particles [117]. These viral particles, however, also activate TLR-independent pathways, such as DNA sensing cytosolic PRRs, if they localize in the cytoplasm instead of the endosome after uptake by cDCs [117]. Receptor agonism by AIC649 then leads to the direct induction of type-I IFNs, in addition to TNF-α and IL-12, and to the indirect secretion of IFN-γ by NK-cells and/or pre-activated T-cells [118]. This activation of innate and adaptive immune responses by AIC649 subsequently results in an antiviral effect against chronic infections with rather diverse viruses, including herpes simplex virus type 1 (HSV-1), hepatitis C virus (HCV), and HBV/WHV [118,119]. For example, twice-weekly administration of peritoneal AIC649 for four weeks to HBV transgenic mice moderately reduced serum viremia that was comparable to oral TDF treatment [120]. Furthermore, a pilot study of twice-weekly administration of intramuscular (im) AIC649 for eight weeks to woodchucks with CHB achieved modest reductions in serum viremia and antigenemia [120]. This study also revealed a unique, biphasic pattern of changes in serum WHV DNA and WHsAg in response to AIC649, with an initial increase in these viral markers after two to four weeks of treatment, and a reduction thereafter that continued after drug withdrawal. The declines in antigenemia, however, did not result into seroconversion to anti-WHs antibodies, most likely due to still detectable WHsAg in serum. For a further delineation of the antiviral potential of AIC649, a recent study in woodchucks with CHB tested a modified treatment regimen, including different administration routes and a longer treatment duration [85]. AIC649 was initially administered iv twice-weekly during twelve weeks of active treatment and then im twice-weekly during nine weeks of maintenance treatment to one group of woodchucks. The AIC649 dose provided consisted of a high number of chemically inactivated parapoxvirus ovis particles of strain NZ-2. Another woodchuck group received iv AIC694 together with daily, oral ETV during the active treatment period that was followed by im administration of AIC649 alone during the maintenance treatment period. Starting between four and seven weeks of AIC649 monotherapy, modest but significant reductions in serum viremia and antigenemia were noted. The achieved declines were different to AIC649/ETV combination treatment that markedly reduced serum WHV DNA, WHsAg, and WHeAg. Serum WHV markers here started to significantly decline between weeks one and ten of combination treatment and stayed suppressed or undetectable after ETV withdrawal in most animals. The antiviral effect during AIC649 monotherapy was again associated with a biphasic response pattern. Different to the previous pilot study, however, seroconversion to anti-WHs antibodies occurred in a subset of woodchucks with more pronounced and sustained declines in antigenemia during AIC649 mono and combination treatment, while anti-WHe antibodies emerged only in a few animals treated with AIC649 and ETV together. Correlating with the changes in serum WHV markers, intrahepatic expression of type-I and type-II IFNs was transiently upregulated during AIC649 monotreatment, and the increase was more pronounced and longer-lasting during combination treatment. Transcription of IFN-α, IFN-β, and IFN-γ in the liver of woodchucks with an SVR after ETV withdrawal correlated with the suppression of intrahepatic WHV replication, including declines in or even undetectable WHV DNA and RNA molecules. AIC649, alone and together with ETV, was safe and well-tolerated by woodchucks based on body weights, body temperatures and hematology and clinical chemistry parameters. Liver enzymes were rarely modulated and the minor elevations in ALT, AST, and SDH during the active treatment period occurred mainly in animals with an SVR during AIC649/ETV combination treatment. Together with the intrahepatic IFN induction, this suggested that the antiviral effect of AIC649 is mediated by non-cytolytic and cytolytic mechanisms shortly after treatment initiation, in addition to the emergence of virus-specific antibodies. Altogether, and as noted before for CpG 21798, AIC649 administration in the setting of low viremia induced by parallel NA treatment enhanced the antiviral effect of TLR9 agonism in woodchucks. Due to the encouraging safety and antiviral efficacy achieved with AIC649 in preclinical animal models of HBV, the immunomodulator was evaluated in patients with CHB during a phase I clinical trial (see below).

### 5.5. Cytoplasmic PRR Agonists

Agonists of cytosolic viral RNA sensing receptors: In addition to TLRs, cytosolic PRRs sensing viral ss and ds RNA, such as RIG-I and nucleotide binding oligomerization domain containing 2 (NOD2), have recently been explored as targets for antiviral therapy [121,122]. SB 9200, an oral prodrug of the dinucleotide SB 9000, was developed by Spring Bank Pharmaceuticals for the treatment of CHB. The dual function of this agonist, including a direct antiviral effect on HBV DNA synthesis by interference of the viral polymerase to engage with pgRNA via SB 9200-activated RIG-I and NOD2 receptors, as well as an immunomodulatory effect via the production of type-I and type-III IFNs by these stimulated receptors, contribute towards the compound’s overall potential against HBV. The initial work in cell culture systems [123,124,125] and in an HBV transgenic mouse model [126] demonstrated marked antiviral effects of SB 9200, and thus justified a further evaluation of this agonist for safety and therapeutic efficacy in the woodchuck. In the initial study, oral SB 9200 was administered daily for twelve weeks at two separate doses to woodchucks with CHB [127]. A dose-dependent and marked reduction in serum WHV DNA and WHsAg was observed, but the antiviral effect was transient and viral relapse occurred after SB 9200 withdrawal. Treatment with the high SB 9200 dose, however, delayed the recrudescence of WHV replication by several weeks. Although antigenemia was significantly reduced from the pretreatment baseline at several time points during and after SB 9200 treatment, WHsAg remained detectable in serum and seroconversion to anti-WHs antibodies was absent in all animals. The transient declines in serum WHV markers correlated with the viral suppression in the liver, including modest declines in WHV DNA molecules, and somewhat more pronounced reductions in WHV RNA species. In addition, the hepatic presence of WHcAg, but not of WHsAg, transiently declined during treatment. A dose-dependent, transient elevation in the liver enzyme SDH was noted in most woodchucks at the time of initial reduction in WHV replication by SB 9200, but levels normalized thereafter, as also indicated by the reduced liver inflammation at the end of treatment. The transaminase rise was associated with a dose-dependent upregulation in the expression of IFN-α, IFN-β, ISGs, such as OAS1, ISG15, and CXCL10, and the pro-inflammatory cytokine, IL-6, in both the periphery and liver. Receptor and ISG transcription stayed elevated in most woodchucks even after drug withdrawal, and this correlated with an increased and prolonged presence of RIG-I and NOD2 receptors in the liver for at least eight weeks. Together with the increased expression of molecules involved in the receptor downstream signaling pathways, including stimulator of interferon genes (STING) and interferon regulatory factor 3 (IRF3), this indicated on-target activation of RIG-I and NOD2 by SB 9200, and that the antiviral effect was most likely based on the non-cytolytic and cytolytic functions of intrahepatic immune cells induced during treatment. SB 9200 administration was well-tolerated at the applied doses, and marked changes in body weight, body temperature, and in hematology and clinical chemistry parameters, other than liver enzymes, were not noted.

In a subsequent study, woodchucks with CHB were sequentially treated with daily, oral SB 9200 for twelve weeks at the high dose and with daily, oral ETV for four weeks [86]. The underlying question here was to determine the antiviral benefit of initial immune response activation via RIG-I and NOD2 receptor agonism followed by NA treatment for further viral suppression *versus* that of NA treatment before immunomodulation, which represents current clinical praxis for pegIFN-α therapy in NA-experienced patients [128]. Thus, one group of woodchucks was administered ETV and then switched to SB 9200 treatment, while another group received SB 9200 followed by ETV treatment. Sequential administration of SB 9200 and ETV produced a more marked reduction in serum WHV markers than the reversed treatment sequence of ETV and SB 9200 that was significant at several timepoints during and after treatment. Correlating with these changes in serum WHV markers, sequential SB 9200/ETV treatment also resulted in more marked declines in intrahepatic viral nucleic acids and antigens, and the effect was most pronounced for WHV RI DNA and cytoplasmic WHsAg. The reductions in viremia and antigenemia in serum and liver, however, were only transient and viral relapse was observed for both treatment regimens after cessation. Recrudescence of WHV replication in woodchucks sequentially treated with SB 9200 and ETV was significantly delayed by several weeks when compared to those sequentially treated with ETV and SB 9200, and serum WHV DNA and WHsAg and intrahepatic WHV DNA and RNA molecules in these animals stayed suppressed below the pretreatment baseline for at least eight weeks. Although marked declines in antigenemia were achieved during treatment, none of the animals lost serum WHsAg or seroconverted to anti-WHs antibodies. Both treatment regimens slowed down the progression of liver disease during the study, including bile duct proliferation and steatosis, and transiently reduced liver inflammation at the end of treatment. Increases in liver enzymes, especially in AST and SDH, were noted with both treatment regimens. Transaminase elevations remained unchanged after drug withdrawal in woodchucks sequentially treated with ETV and SB 9200, while they reversed after the end of treatment and normalized in animals sequentially treated with SB 9200 and ETV. The initial rise in liver enzymes during the latter treatment regimen correlated with an increased expression of IFN-α, IFN-β, ISG15, CXCL10, and IL-6 in the periphery. In the liver, maximum transcription of type-I IFNs and ISGs was mainly detected after the end of treatment, indicating that the delayed viral rebound after sequential SB 9200/ETV treatment was most likely a result of the induced antiviral immune response. The elevations in type-I IFNs and ISGs, together with the increased transaminases during treatment and absent liver inflammation at the end of treatment, further suggested that the antiviral effect induced by sequential SB 9200/ETV treatment was mediated by non-cytolytic and cytolytic immune mechanisms. As noted before for SB 9200 monotherapy, sequential treatment with SB 9200 and ETV was also well-tolerated by woodchucks. Altogether, these results suggested that the initial induction of an innate immune response with SB 9200 followed by an additional reduction of viral load with ETV resulted in an antiviral effect that was superior over the reversed treatment sequence of ETV and SB 9200. Since the woodchuck study established that immunomodulation with SB 9200 should be initiated in NA treatment-naïve rather than -experienced patients with CHB for achieving a superior antiviral effect, this strategy was subsequently tested and confirmed in a phase II clinical trial, demonstrating the capacity of the woodchuck animal model to predict the treatment outcome to RIG-I and NOD2 receptor agonism in humans (see below).

Agonists of cytosolic viral DNA sensing receptors: In recent years, the role of PRRs in sensing HBV infection has been explored beyond the family of TLRs and RIG-I like receptors (RLRs). For example, the cytosolic viral DNA sensing receptor, cGAS, was shown to be activated in vitro by artificially high concentrations of HBV DNA but not by viral RNA [70]. Another in vitro study demonstrated that IFN-γ inducible protein 16 (IFI16) located within the nucleus of human hepatoma cells recognizes HBV cccDNA and that overexpression of this receptor affects viral replication [129]. In addition, the antiviral potential of STING agonists was demonstrated in vivo in a mouse model following hydrodynamic injection of a plasmid DNA encoding for HBV [130]. Besides these PRRs, the involvement of other cytosolic viral DNA sensing receptors (CDSs) and inflammasome-forming complexes was recently studied in woodchucks experimentally infected with WHV [71]. The study investigated the kinetics of sixteen PRRs from different families, including TLRs, CDSs, RLRs, nucleotide-binding and oligomerization domain NOD-like receptors (NLRs), and inflammasomes. While the intrahepatic expression of all PRRs was unchanged five weeks after WHV inoculation, maximum transcription of most receptors was observed during AHB and subsequent viral resolution. CDSs, such as IFI16 and Z-DNA binding protein 1 (ZBP1) or DNA-dependent activator of IFN-regulatory factors (DAI), and inflammasomes, including absent in melanoma 2 (AIM2) and NLR family pyrin domain containing 3 (NLRP3), displayed comparable or even higher mRNA and/or protein levels, when compared to other more well-characterized PRRs, such as TLRs and RLRs [131,132,133]. These results prompted a follow-up study for evaluating in vitro the antiviral effect associated with agonism of these receptors during chronic WHV infection. Agonistic stimulation of IFI16, ZBP1/DAI, and AIM2 receptors in primary hepatocyte cultures generated from woodchucks with CHB demonstrated an activation of the receptor downstream signaling pathways that was associated with a strong antiviral effect on WHV replication and virion secretion [134]. Mirroring the broad in vivo activation of numerous intrahepatic PRRs in woodchucks during AHB and WHV resolution, parallel agonistic stimulation of all three receptors significantly enhanced the antiviral effect in vitro. Liver-targeted, single dose iv administration of an agonist of the ZBP1/DAI and AIM2 receptors within a lipid carrier to WHV-naïve woodchucks stimulated both PRRs and activated their respective downstream signaling pathways, leading to the expression of the effector cytokines, IFN-β or IL-1β and IL-18, respectively [134]. Following PRR stimulation, PBMCs of these animals presented with increased IFN-γ secretion, most likely via the activation of peripheral T-cells by the induced IL-18. Receptor agonism was safe and did not affect body weight, body temperature, and hematology and clinical chemistry parameters. Only minor increases in SDH but not in other liver enzymes were noted that correlated well with absent changes in liver inflammation. Future studies will need to address the safety and antiviral potential of repeat agonistic stimulation of ZBP1/DAI and AIM2 and comparable receptors in the liver of woodchucks with CHB.

### 5.6. Checkpoint Inhibitor

Virus-specific T-cell responses are critical for the immunological control and clearance of HBV infection. In patients with CHB, exhausted T-cells that are characterized by poor cytotoxic effector functions and impaired cytokine production present with sustained expression of inhibitory receptors, including programmed cell death protein 1 (PD-1), lymphocyte-activation gene 3 (LAG-3), cytotoxic T-lymphocyte antigen 4 (CTLA4), and T-cell immunoglobulin and mucin domain-containing protein 3 (TIM3) [135]. Thus, blocking the interaction of PD-1 with its ligands (PD-L1 or 2) by antibodies may be beneficial in recovering the antiviral effector functions of HBV-specific T-cells [136]. The chimeric, monoclonal antibody (mAb) wc6D5 was developed by Bristol Myers Squibb for targeting woodchuck PD-L1, which resembles the human anti-PD-L1 antibody, BMS 936559, used in the anti-cancer treatment of patients [87]. Woodchucks with CHB were iv treated four times with either vehicle control or wc6D5 administered every second day over ten days [87]. While the mAb plasma titers reached a saturation level during the four-dose treatment regimen, a transient antiviral effect was only achieved in one woodchuck that received wc6D5. The sole responder animal experienced a modest decline in serum WHsAg three weeks after the mAb administration that was more pronounced than the parallel reduction in WHV DNA, along with a minor but notable increase in the liver enzyme ALT. Since most mAb-treated woodchucks had high levels of viremia and antigenemia at pretreatment, these animals presented with relatively low expression levels of PD-1 and PD-L1 on peripheral T-cells or intrahepatic antigen presenting cells (APCs), respectively, similar to findings in patients with CHB [137]. The study also determined PD-1 expression on peripheral CD4+ and CD8+ T-cells (i.e., the latter cells were characterized as CD3+ CD4− cells in the absence of an antibody against woodchuck CD8) of WHV-uninfected and experimentally and naturally WHV-infected woodchucks. Compared to WHV-naïve woodchucks, experimentally infected animals did not have elevations in PD-1 expression on CD8+ T-cells that was different to naturally infected animals, with significant higher receptor transcription. PD-1 expression on CD4+ T-cells, however, was not different between the three groups. Based on these results, naturally WHV-infected woodchucks were enrolled in a subsequent study for evaluating the antiviral potential of wc6D5 in combination with ETV. Animals were treated daily with oral ETV for twelve weeks. Starting in week six, woodchucks then received four iv administrations of either the wc6D5 mAb or a control isotype antibody provided every third to fourth day over a ten-day period. As expected, combination treatment with ETV and the control antibody produced a marked but transient effect on WHV DNA but not on WHsAg in serum. This was different to woodchucks that received ETV in combination with wc6D5 and that achieved a marked decline in serum WHsAg within three weeks after the initiation of mAb treatment. Consequently, a subset of animals administered the combination of ETV and wc6D5 was characterized as responders, based on the long-lasting treatment response after ETV withdrawal, including two woodchucks with sustained suppression of serum WHV DNA, WHsAg, and WHeAg that lasted for at least ten weeks. However, none of the responder woodchucks with an SVR seroconverted to anti-WHs antibodies. Contrary to the initial study in experimentally WHV-infected woodchucks, naturally WHV-infected responder animals of the second study had no marked elevation in liver enzymes. Combination treatment with ETV and wc6D5 was safe and well-tolerated, as changes in hematology and clinical chemistry parameters were absent, indicating that the antiviral effect was mainly based on the non-cytolytic functions of T-cells activated by PD-1/PD-L1 blockade. In an attempt to identify biomarkers predictive of the response to wc6D5 administration, viremia and antigenemia levels in serum, transaminase levels, presence of PD-L1 on APCs and numbers of intrahepatic CD3+ T-cells and Kupffer cells, IFN-γ secretion of WHV-specific T-cells, and PD-1 expression on CD8+ T-cells in blood at pretreatment were compared between responder and non-responder woodchucks of the second study. Among all viral markers and host factors tested, it appeared that low pretreatment serum levels of WHsAg are most predictive of an antiviral response to PD-1/PD-L1 blockade by mAb treatment. BMS 936559 has not been clinically tested in patients with CHB so far.

### 5.7. Therapeutic Vaccination

Therapeutic vaccines against CHB have been applied to animal models and patients to enhance the host cellular immune response to viral antigens. However, therapeutic vaccination has only been limitedly successful for several reasons, including the high viral antigen load at the time of vaccine administration and a lack of effector T-cell expansion following vaccination [138]. Application of the woodchuck for testing the potential of therapeutic vaccines using rather diverse approaches, such as protein- and DNA-based vaccines containing or encoding for WHcAg and/or WHsAg, respectively, alone and in combination with a T_H_ cell peptide and other adjuvants, and during NA treatment with clevudine (CLV) and lamivudine (LAM), was summarized previously [139]. Although these studies often failed to demonstrate antiviral efficacy, they nevertheless highlighted the importance of timing and duration of the applied therapeutic vaccination regimens, and the benefit of combining such vaccines with NA treatment for lowering the viral (and antigen) load. Furthermore, a prime-boost immunization strategy using plasmid DNA and an adenoviral vector for the expression of WHsAg and WHcAg, alone and in combination with ETV, has been evaluated in the woodchuck [140]. Compared to ETV monotherapy, therapeutic DNA vaccination in combination with the NA resulted in a prolonged suppression of serum viremia and antigenemia in woodchucks with CHB, and a subset of these animals also developed anti-WHs antibodies. A more recent study tested therapeutic DNA vaccination together with ETV treatment and antibody-mediated blockage of PD-1/PD-L1 interaction in woodchucks with CHB [88]. Animals receiving the triple combination regimen were treated with sc ETV for 28 weeks for an initial reduction of WHV DNA. ETV was firstly administered via osmotic pumps for twelve weeks and, starting in week ten, was subsequently provided by weekly sc injections. Starting in week twelve, and following a single, im cardiotoxin administration, weekly im injections of plasmid DNA encoding WHcAg and WHsAg were given for twelve weeks. This was followed by three iv administrations of a commercially available rabbit polyclonal antibody against PD-L1 provided every second day during week 24 of treatment. Control groups received either placebo, ETV, or ETV and DNA vaccine. Compared to mono and double treatment, the triple combination treatment regimen enhanced the antiviral effect and resulted in undetectable serum WHV DNA, low-level or loss of serum WHsAg, and strongly reduced or led to undetectable intrahepatic WHV RI-DNA and cccDNA in a subset of woodchucks. These animals also seroconverted to anti-WHs antibodies that remained detectable for at least 14 weeks. Woodchucks undergoing mono or double treatment achieved only transient reductions in circulating and intrahepatic WHV markers, had no anti-WHs antibodies, and viral relapse occurred in all animals after treatment cessation. In woodchucks treated with the triple combination regimen, an increase in the degranulation function of peripheral WHV-specific CD8+ (i.e., CD3+/CD4−) T-cells was observed immediately after the administration of the anti-PD-L1 antibody, and the improvement was more pronounced for WHcAg- than for WHsAg-specific CD8+ T-cells. In addition, a strong proliferation of T-cells to stimulation with peptides covering the WHcAg was present after the antibody administration. While a transient increase in the liver enzyme AST was observed in most ETV-treated woodchucks at the time of reductions in serum WHV DNA, a similar transaminase rise was absent during or after the antibody administration. Altogether, these results indicated that the retrieval of mainly non-cytolytic T-cell functions by PD-1/PD-L1 blockade contributed to a sustained immunological control of WHV but that therapeutic (DNA) vaccination was essential for the induction of virus-specific CD8+ T-cells. While these results are encouraging towards exploring therapeutic vaccination regimens in greater detail, follow-up studies in a larger woodchuck cohort are needed to confirm these findings.

## 6. Evaluation of Novel Host and Viral Targets in Woodchucks

Besides inhibiting the function of the HBV polymerase with NAs, novel compounds targeting other steps of the viral life cycle have been developed. In vitro knockdown of WHV by siRNAs targeting the surface, core and x regions of the viral genome in primary hepatocyte cultures generated from woodchucks with CHB resulted in a marked, transient decline of viral transcription and replication and virion secretion [141]. In addition, RNA interference increased the expression of ISGs via IFN-β produced by pathways involving TLRs and protein kinase R (PKR) that was independent of WHV gene silencing [142]. While RNA interference was not assessed in vivo yet, other antivirals inhibiting cell entry, gene transcription, and antigen release were recently evaluated in woodchucks (Figure 2 and Table 1).

### 6.1. Entry Inhibition

hzVSF: Restricting viral entry into susceptible cells by blocking the interaction of the virus with its host receptor(s) is another treatment strategy tested previously for HBV [143]. Myrcludex B (Hepcludex), a synthetic, myristolated peptide derived from the preS1 domain of the L-HBsAg that interacts with the NTCP receptor, was the first-in-class entry inhibitor developed against infections with HBV and hepatitis delta virus (HDV). This peptide inhibits mainly de-novo HBV infection, as determined in a preclinical mouse study [144], and is currently assessed in clinical phase III trials in HBV-infected patients and in patients super-/co-infected with HDV [145]. In a recent woodchuck study, another entry inhibitor with a novel mechanism of action was evaluated for safety and efficacy against WHV [72]. This entry inhibitor developed by ImmuneMed, also known as humanized virus suppressing factor (hzVSF), is a mAB targeting vimentin that is upregulated after infection with various viruses on the cell surface. Antibody binding then mediates an antiviral effect and reduces inflammation induced by the human coronaviruses OC43 and SARS-CoV-2 [146,147]. Another study demonstrated in vitro that hzVSF inhibits endocytosis-based HBV entry via the NTCP receptor by binding to virus-induced surface vimentin, thereby likely inducing changes in the intracellular vimentin localization [148]. The study in woodchucks revealed that cell surface vimentin in the liver is strongly upregulated during WHV infection, similar to patients with HBV infection [72]. After confirming the cross-reactivity of hzVSF to woodchuck vimentin, a safety and dose finding study was performed in WHV-uninfected and -infected animals. Twice-weekly iv injection of hzVSF at a high dose for twelve weeks into WHV-naive woodchucks was safe and well-tolerated, when compared to placebo-treated animals, and no significant changes in body weight, body temperature, hematology and clinical chemistry parameters, including liver enzymes, and organ histopathology were noted. Repeat iv administration of hzVSF twice-weekly for twelve weeks to woodchucks with CHB at a low, intermediate, and the high dose was subsequently tested to determine the antiviral efficacy of the entry inhibitor. Serum viremia and antigenemia modestly declined during treatment, and the reductions were largely dose-dependent and only significant with the high hzVSF dose, when compared to the pretreatment baseline. A marked antiviral effect was achieved with the intermediate hzVSF dose in one woodchuck with relatively low baseline WHV markers. This particular animal presented with undetectable serum WHV DNA and WHsAg during treatment, and the SVR after drug withdrawal lasted at least for four weeks. In all other animals, viral relapse occurred immediately after treatment discontinuation. Based on the sole responder woodchuck, indicating a benefit of low WHV replication at treatment initiation, a follow-up study in woodchucks with CHB tested iv administration of hzVSF twice-weekly for twelve weeks at the intermediate dose in combination with oral TAF provided daily. Contrary to hzVSF monotreatment, combination treatment with the NA demonstrated an enhanced antiviral effect, as serum viremia and antigenemia declined faster and at a greater magnitude, and the reductions were also superior over TAF monotherapy (see above). Noticeably, serum WHV markers stayed suppressed or undetectable in half of the woodchucks after treatment discontinuation, while viral relapse was delayed in the other half of animals, when compared to monotherapy with hzVSF or TAF. Furthermore, intrahepatic WHV DNA and RNA molecules significantly declined during combination *versus* mono treatment, and were durably reduced or even undetectable in woodchucks with an SVR. WHcAg and WHsAg presence in the liver of these animals showed a similar pattern of decline. Correlating with the profound antigenemia reduction in blood and liver, seroconversion to anti-WHs and/or anti-WHe antibodies occurred in woodchucks with an SVR after monotherapy with the intermediate hzVSF dose, alone and together with TAF. hzVSF treatment in woodchucks with CHB was safe and well-tolerated based on the same parameters applied for WHV-naïve woodchucks. In addition, hzVSF treatment with the high dose, and especially with the intermediate dose in combination with TAF, transiently reduced liver inflammation, which was associated with parallel declines in Kupffer cells. These reductions correlated with lower intrahepatic CD3+ T-cell accumulation and liver replenishment by new hepatocytes. They further correlated with largely unchanged transaminases, although woodchucks with an SVR had a more pronounced but transient elevation in the liver enzyme SDH during treatment. Altogether, this indicated viral entry inhibition rather than activation of cytolytic NK- and T-cells by hzVSF as the underlying antiviral mechanism, in addition to the induced antibody response. This mechanism of action of hzVSF that is different to Myrcludex B may be advantageous in the treatment of patients. The humanized mAb does not directly block the NTCP receptor, which can affect the transport of conjugated bile acids, nor is expected to induce an anti-drug antibody reaction during repeat administration. Prolonged inhibition of HBV entry, and thus viral re-infection, by targeting surface vimentin on infected hepatocytes may allow to lower the persistent cccDNA pool in the infected liver over time, as shown for Myrcludex B in the humanized chimeric mouse model of HBV [144]. Overall, the results in the woodchuck suggest a therapeutic potential of hzVSF against HBV, and probably against HDV, especially if provided as an add-on to NA treatment. hzVSF has not been clinically tested in patients with HBV/HDV infection so far.

### 6.2. Surface Inhibition

The prolonged exposure to abundant HBV proteins, such as HBsAg, in blood and liver contributes to the dysfunctional antiviral immune response in patients with CHB, and is thought to be responsible for the impaired functions of APCs, as well as the tolerizing (i.e., exhausting) effect on HBV-specific T- and B-cells [53,54,55,56,64]. Thus, reducing antigenemia should aid in the retrieval of effective innate and adaptive immune responses against HBV. For achieving functional cure in patients, sustained loss of HBsAg is considered a prerequisite for the immunological control of HBV in regard to the induction of virus-neutralizing antibodies [149].

RG7834: This small molecule inhibitor of HBV gene expression was developed by F. Hoffmann-La Roche for reducing the HBsAg level in patients [150]. RG7834 targets the host noncanonical poly(A) polymerases PAPD5 and PAPD7 that stabilize HBV RNA via the interaction with the viral posttranscriptional regulatory element, and thus reduces HBV sub-genomic RNA transcripts and increases viral RNA degradation. In the humanized chimeric mouse model of HBV [151], RG7834 monotherapy significantly reduced HBV DNA and HBsAg in serum, albeit to still detectable levels, but the antiviral effect was enhanced by combination treatment with ETV and/or pegIFN-α. The therapeutic efficacy of RG7834 was further evaluated in woodchucks with CHB, alone and together with ETV and/or wIFN-α [74]. Groups of woodchucks were treated for 14 weeks with oral RG7834 provided twice-daily, with oral ETV administered daily, and/or with sc wIFN-α given initially three times per week and then twice-weekly after a 16-day treatment interruption due to IFN-related adverse effects. Administration of RG7834, alone and in combination with ETV and/or wIFN-α significantly reduced serum WHV DNA and WHsAg from the pretreatment baseline. The reductions in serum viremia, and especially in antigenemia, during combination treatment were greater than those achieved by monotreatment. The greatest decline, however, was achieved by treatment with the triple combination of RG7834, ETV, and wIFN-α. Correlating with the antiviral effect, the treatment duration required for an average reduction of WHsAg by at least 1.5 log_10_ from the pretreatment baseline varied substantially between the treatment regimens. For example, woodchucks administered RG7834 achieved this decline in antigenemia level within 20 days of treatment, while animals provided ETV needed much longer and reached this endpoint after 76 days. Contrary, in woodchucks administered RG7834, ETV, and wIFN-α, this duration was significantly shortened to six days, indicating an antiviral benefit of the triple combination treatment. Similar to the changes in serum WHV markers, maximum and significant reductions in intrahepatic viral nucleic acids occurred earlier, were more pronounced, and longer-lasting (at least for WHV cccDNA) with the triple combination than with any other treatment regimen. While RG7834 and ETV monotherapy mainly effected surface mRNA or pgRNA, respectively, RG7834, ETV, and wIFN-α combination treatment significantly reduced both RNA molecules. Furthermore, the reductions in WHsAg (and, although not determined, most likely in WHcAg) achieved by the triple combination treatment were sufficient to induce the proliferation of T-cells to stimulation with WHV antigen-specific peptides, and this response, albeit transient, was inversely correlated with the antigenemia load. Despite the marked reductions in serum WHsAg level, seroconversion to anti-WHs antibodies was absent, and viral relapse occurred in all animals after treatment discontinuation. RG7834 administration, alone and together with ETV (but not with wIFN-α), was safe and well-tolerated by woodchucks, and did not markedly elevate the liver enzymes ALT, AST, and SDH during treatment, indicating that the RG7834-induced antiviral response was likely mediated by the non-cytolytic activity of intrahepatic NK- and/or T-cells. Since the triple combination regimen resulted in the most profound antiviral effect, this suggested that extending the treatment duration with RG7834 and ETV (and possibly IFN-α) beyond 14 weeks may be able to durably suppress HBV in patients. However, due to undisclosed toxicity mediated by RG7834 [152] during a phase I clinical trial, which was most likely associated with the blood-brain barrier penetration of the compound, further development has been halted.

REP 2055 and REP 2139: These two nucleic acid polymers (NAPs) are phosphorothioate ODNs developed by Replicor for blocking the release of HBsAg derived from HBV cccDNA and integrated viral DNA in hepatocytes thereby reducing the antigenemia level in patients and allowing host mediated clearance of the virus [153]. In Pekin ducks infected with duck hepatitis virus (DHBV) and in humans infected with HBV and/or HDV, both NAPs selectively target the assembly of subviral particles (SVPs) resulting in a multilog reduction of circulating HBsAg or even loss of this antigen in subsets of animals and patients [154,155,156,157]. Clearance of HBsAg during NAP monotherapy is usually accompanied by undetectable viral DNA in serum and/or seroconversion to virus-neutralizing antibodies (i.e., SVR or functional cure). The therapeutic efficacy of the clinically active NAPs, REP 2055 and REP 2139, was also evaluated in woodchucks with CHB as single agents [75]. In the first study, groups of woodchucks received sc administrations of either REP 2055 or REP 2139 for three weeks provided thrice-weekly. In the second study, animals received three times per week sc administrations of REP 2139 in form of a calcium chelate complex (REP 2139-Ca) at a 1.5-fold higher dose for five weeks. In contrast to ducks and humans, but comparable to HBV transgenic and humanized chimeric mice, monotherapy with REP 2055 and REP 2139-Ca (but not with REP 2139) only modestly reduced serum WHsAg in a subset of woodchucks. Antigenemia modulation in these animals occurred without parallel declines in serum WHV DNA or increases in the liver enzyme AST. The weak or absent antiviral effect in rodent models of HBV, despite an NAP accumulation in the liver that was comparable to those achieved in ducks and patients with REP 2055 and REP 2139, suggested different morphogenesis and/or secretion of SVPs in infected hepatocytes of these animals.

## 7. Significance of Preclinical Woodchuck Studies for Clinical Trials in Patients

The preclinical studies in woodchucks summarized above were critical in determining the therapeutic efficacy and, more importantly, the safety of novel antivirals that subsequently justified their assessment in patients. Among the TLR7 agonists evaluated so far in the woodchuck, only GS-9620 (Vesatolimod) has been tested in several clinical trials. In healthy volunteers, oral administration of a single, accelerating GS-9620 dose induced a dose-dependent, transient induction of ISG15 in blood without any detectable INF-α in serum [98]. In two phase Ib clinical trials, NA treatment-naïve (NCT01590641) and -experienced patients with CHB (NCT01590654) were administered oral GS-9620 as a single, increasing dose or as two doses one week apart. At the applied dose range, no significant changes in serum HBV DNA and HBsAg were observed, but a dose-dependent ISG15 induction in blood and increased IP-10/CXCL10 levels in serum were noted, again without detectable serum IFN-α in most patients [100,101]. Single or repeat GS-9620 dosing in these studies was safe and well-tolerated by patients, and without significant adverse events or laboratory abnormalities, including changes in liver enzymes or platelet counts. Similar results were obtained in phase II clinical trials in NA treatment-naïve (NCT02579382) and -experienced patients with CHB (NCT02166047), with no significant changes in serum HBsAg, even with the highest oral GS-9620 dose given weekly for up to twelve weeks, alone and in combination with TDF [101,102]. Higher GS-9620 doses were not tested in patients, but two individuals who inadvertently received doses exceeding the highest dose by 2.5- or 5-fold on the first day of treatment experienced severe side effects requiring hospitalization, possibly due to drug tolerability issues [101]. In addition to a dose-dependent ISG induction in blood [101,102], PBMCs from virologically suppressed patients undergoing GS-9620 treatment demonstrated a functional restoration of HBV-specific T-cells and increased activation and function of NK-cells, including a lower ability to suppress T-cell responses [158]. As already mentioned, a recent analysis of the different treatment outcomes in chimpanzees, woodchucks, and patients revealed that GS-9620 was less selective for woodchuck TLR7 and additionally activated TLR8 at high dosage that may explain the superior antiviral effect achieved in this animal model [83]. Furthermore, while oral GS-9620 treatment of woodchucks with CHB at an approximately 8- to 16-fold lower dose than those applied in the initial efficacy study [80] was unable to produce an antiviral effect, as observed in patients with CHB, it nevertheless resulted in ISG15 expression in blood without parallel induction of IFN-α and IL-12 in serum of WHV-naïve animals [83] that was comparable to the PD response in healthy human volunteers and in patients treated with the highest and tolerated dose of the TLR7 agonist [98,100,102]. Overall, these results confirmed the reproducibility of the GS-9620 mediated treatment outcome in woodchucks and humans at low dosage. The results further indicated the need to check for unwanted off-target effects of novel immunomodulators provided at high dosage during preclinical studies in animal models that may later affect their tolerability in patients.

The TLR8 agonist, GS-9688 (Selgantolimod), was first evaluated in phase Ib clinical trials in healthy human volunteers (ACTRN12616001646437) and in virally suppressed patients with CHB (ACTRN12617000235303). Single, accelerating doses of oral GS-9688 were safe and well-tolerated and induced a dose-dependent induction of IL-12p40 and interleukin-1 receptor antagonist (IL-1RA), as well as other cytokines, chemokines, and acute phase proteins in serum of uninfected individuals [159]. Thereafter, patients on NA treatment received weekly oral GS-9688 at two different doses for two to four weeks, while patients without prior NA treatment received weekly the higher GS-9688 dose for two weeks [160]. GS-9688 treatment was safe and well-tolerated by patients and induced transient, dose-dependent increases in the serum levels of IL-12p40 and IL-1RA; however, the strong cytokine induction was not associated with an antiviral effect. While the increase in serum IL-12p40 in humans confirmed the PD results in woodchucks [83], the difference in the treatment outcome, especially between woodchucks and NA treatment-naïve patients, is most likely due to lower GS-9688 dosage and treatment duration. The phase II clinical trials of GS-9688 in both NA treatment-naïve (NCT3615066) and -experienced patients (NCT03491553) have recently been completed. A subset of viremic patients treated weekly with increasing GS-9688 doses for 24 weeks in combination with daily TAF, achieved a decline in HBsAg level of >0.5 log_10_ that was sustained for at least 24 weeks [161]. In addition, a subset of virally suppressed patients treated with GS-9688 using the same dose range, dosing frequency, and treatment duration experienced a loss of HBsAg and/or HBeAg 24 weeks after drug withdrawal [162]. GS-9688 treatment was safe and well-tolerated by all patients and induced again a dose-dependent cytokine response. Another phase II clinical trial with GS-9688 in combination with TAF, siRNA, and/or an anti-PD-1 mAb (NCT04891770) is expected to be initiated soon.

In regard to the TLR9 agonist AIC649, a phase I clinical trial tested single, ascending iv doses for safety and PD response in NA treatment-naïve and -experienced patients with CHB [163]. While treatment-associated tolerability issues were not noted, significant reductions in the serum levels of HBV DNA and HBsAg were absent in most patients. However, one individual treated with an intermediate AIC649 dose had a decline in HBV DNA twelve weeks after the administration. This change in viremia was associated with HBeAg loss and seroconversion to anti-HBe antibodies, and the patient also became transiently positive for anti-HBs antibodies despite unchanged HBsAg levels. Furthermore, AIC649 induced changes in the innate immune response, with transient increases in IL-1β, IL-6, IL-8, and IFN-γ, and decreases in IL-10 in the serum of patients, especially of those who received the highest dose of the TLR9 agonist that is equal to the dose repeatedly administered to woodchucks with CHB [85]. A phase Ib/IIa clinical trial of AIC649 is planned.

The two studies performed with SB 9200 (Inarigivir) in woodchucks [86,127], especially the one that tested sequential treatment of the RIG-I/NOD2 agonist with ETV, were critical in designing a suitable treatment regimen for the subsequent phase IIa clinical trial in patients with CHB. In NA treatment-naïve patients, a dose-dependent reduction in serum HBV DNA, particularly in HBeAg-negative patients, was noted following weekly administrations of oral SB 9200 at increasing doses for twelve weeks (NCT02751996) [164]. Add-on administration of daily, oral TDF after the end of SB 9200 treatment further resulted in a >0.5 log_10_ reduction in serum HBsAg in approximately one-fourth of patients [164]. The highest SB 9200 dose that was intended for administration to patients on a daily base or thrice-weekly for six weeks (NCT03932513) is comparable to the low SB 9200 dose tested in woodchucks. The clinical trial, however, was terminated when severe adverse effects, including one mortality, were observed in patients after parallel, daily administration of this SB 9200 dose together with oral TAF (NCT03434353) [165]. Of note is that the treatment regimen is different to the sequential treatment regimens previously applied to woodchucks and subsequently assessed in patients, and that the combination of SB 9200 and TAF was never preclinically tested. Overall, these results demonstrated the duplicability of the outcome of sequential treatment with SB 9200 and NAs in woodchucks and humans. However, the outcome of parallel treatment with SB 9200 and TAF in patients indicated the need to evaluate even slightly modified treatment regimens involving novel immunomodulators and NAs for safety in preclinical studies in animal models of HBV before their application to humans.

## 8. Conclusions

One area of current development of novel therapeutic treatment options against CHB in patients is focused on immunotherapy. A prerequisite for modulating the anti-HBV immune response is to have preclinical animal models available that are immunocompetent and outbred and that closely model the immunopathogenesis of HBV infection in humans. For several decades, woodchucks with CHB have been a reliable animal model for testing safety, PK and PD parameters, and antiviral efficacy of novel compounds, including DAAs, immunomodulators, and therapeutic vaccines. Besides serving as a surrogate animal model that supports all steps of the viral life cycle and associated liver disease progression present during CHB in patients, there are several benefits to the use of woodchucks in HBV research. The possibility of collecting serial liver biopsies from the same animal for the determination of virological and immunological markers at the site of viral replication, including viral and host gene expression and protein presence and associated liver inflammation and injury, is undoubtedly the main advantage of this model for drug development and evaluation, when compared to the various mouse models of HBV. Although recent developments in obtaining fine needle aspirates have provided a less-invasive procedure to collect liver samples from patients, new and highly advanced technologies are needed for simultaneously measurement of several parameters from a tissue of very small size [166]. The rather long durations of treatment and follow-up after drug withdrawal during preclinical studies in the woodchuck are comparable to those in patients, and thus are critical for determining any SVR to treatment (e.g., induction of functional cure, delay or prevention of liver disease progression and HCC development) and possible adverse effects mediated by novel drugs and drug combinations. Unlike mice in which blood collection is limited, individual woodchucks can be frequently bled (i.e., daily or weekly) and blood subjected to standardized assays for the measurement of serology, hematology, and clinical chemistry parameters, including viremia and antigenemia levels, antibody titers, host gene expression, immune cell functions, and liver enzymes, that all have been established for this animal model. Comparison of these measurements is useful in the evaluation of safety and therapeutic efficacy of novel compounds that belong to the same family of drugs and/or have a comparable mechanism of action (e.g., NAs and TLRs). While host-specific reagents for performing most protein-based assays are still lacking for the woodchuck, despite its use in HBV research for nearly 40 years, the identification of cross-reactive antibodies in recent years has overcome certain challenges relating to assays based on immunohistochemistry or fluorescence activated cell sorting. Thus, the continuation of preclinical studies in the woodchuck, as summarized in this article, will tremendously support the current efforts of academic groups and pharmaceutical companies in the pursuit of a much-needed cure for the worldwide 296 million patients with CHB. As established for all NAs, as well as for systemic IFN-α and several PRR agonists, the results obtained in the woodchuck are predictive of the PD response and treatment outcome in patients with CHB.

## Figures and Tables

**Table 1:** NAs and DAAs recently evaluated in the woodchuck for safety and antiviral efficacy against CHB.

Antiviral	Abbreviation/Brand Name	Dose	Group Size (Animal Number)	Treatment/ Follow-Up Duration (Weeks)	Antiviral Effect (Serum)			Treatment Outcome	Additional Results	Adverse Effects	Reference
					WHV DNA (Log Red.)	WHsAg (Log Red.)	WHeAg (ODU Red.)				
NA	TAF/Vemlidy	5 mg/kg (po, QD)	4	12/4	6.38	3.24	1.86	Transient (viral relapse)	Transient red. in: − WHV RI-DNA and cccDNA − SDH	None	[72]
Viral entry inhibitor	Anti-vimentin monoclonal antibody hzVSF	hzVSF (4 mg/kg, iv, BIW) + TAF (5 mg/kg, po, QD)	4	12/4	7.27	3.47	1.87	SVR/Functional cure (seroconversion to anti-WHs and anti-WHe antibodies) in a subset of animals	Sustained red. in: − WHV RI-DNA and cccDNA − surface and pg RNAs − liver inflammation Transient inc. in: − SDH	None	[72]
Gene expression inhibitor	RG7834	RG7834 (10 mg/kg, po, BID) + ETV (0.1 mg/kg, po, QD) + wIFN-α5 (0.1 mg/kg, sc, TIW followed by BIW)	5	14/10	7.46	5.0	-	Transient (viral relapse)	Transient red. in: − WHV RI-DNA and cccDNA − surface and pg RNAs	IFN-related adverse effects	[74]
Antigen release inhibitor	REP 2055/REP 2139-Ca	REP 2055/REP 2139-Ca (10–15 mg/kg, sc, TIW)	2–6	3–5/0–1	0	~0.5	-	-	-	None	[75]

Abbreviations: anti-WHe, antibodies against WHeAg; anti-WHs, antibodies against WHsAg; BID, twice a day; BIW, twice a week; cccDNA, covalently closed circular DNA; ETV, entecavir; hzVSF, humanized virus suppressing factor or viral entry inhibitor, a monoclonal antibody from ImmuneMed; IFN, interferon; inc., increase; iv, intravenous; log, logarithmic; NA, nucleos(t)ide analog; ODU, optical density unit; po, oral; QD, every day; pg, pre-genomic; red., reduction; REP 2055 and REP 2139-Ca, inhibitors of HBV subviral particle assembly and surface antigen release from Replicor; RG7834, inhibitor of HBV gene expression from F. Hoffmann-La Roche; RI-DNA, replicative intermediate DNA; SDH, sorbitol dehydrogenase; sc, subcutaneous; SVR, sustained antiviral response; TAF, tenofovir alafenamide fumarate; TIW, three times a week; WHeAg, WHV e antigen; WHsAg, WHV surface antigen; WHV, woodchuck hepatitis virus; wIFN-α5, recombinant woodchuck IFN-α5.

**Table 2:** Immunomodulators recently evaluated in the woodchuck for safety and antiviral efficacy against CHB.

Immunomodulator	Abbreviation/Brand Name	Dose	Group Size (Animal Number)	Treatment/ Follow-Up Duration (Weeks)	Antiviral Effect (Serum)			Treatment Outcome	Additional Results	Adverse Effects	Reference
					WHV DNA (Log Red.)	WHsAg (Log Red.)	WHeAg (ODU Red.)				
Interferon-alpha	wIFN-α5 (systemic)	20 µg/animal (sc, TIW for 7 weeks) then 100 µg/animal (sc, TIW for 8 weeks)	12	15/8	3.62	2.42	-	Transient (variable viral relapse)	Transient red. in: − WHV RI-DNA and cccDNA − surface and pg RNAs Transient inc. in: − ALT and AST − liver inflammation	None	[78]
	wIFN-α5 (AAV-mediated; fused to apolipoprotein A-I)	5 × 10^12^ vg (iv, once) + ETV (0.5 mg/kg, po, QD)	5	4/12	4.5	0.6	-	Transient (delayed viral relapse compared to control)		None	[79]
TLR7 agonist	GS-9620/Vesatolimod	2.5–5.0 mg/kg (po, QOD, QOD in QOW, or QW)	12	4–8/31	Up to 6.2	Undetectable/Loss	-	SVR/Functional cure (seroconversion to anti-WHs antibodies) in a subset of animals	Sustained red. in: − WHV RI-DNA and cccDNA, − surface and pg RNAs Transient inc. in: − ALT and AST	Thrombocytopenia (reversible)	[80]
	APR002	5–30 mg/kg (po, QW) + ETV (0.1 mg/kg, po, QD)	4	20/16	6.64–7.33	2.40–3.28	2.50–2.77	SVR/Functional cure (seroconversion to anti-WHs and anti-WHe antibodies) in a subset of animals	Sustained red. in: − WHV RI-DNA and cccDNA, − surface and pg RNAs Transient inc. in: − ALT, AST, and SDH	Hypothermia (reversible)	[81]
	RG7854	30–120 mg/kg (po, QOD)	5–6	14–24/11	2.43–5.14	2.60–2.87	1.40–1.53	SVR/Functional cure (seroconversion to anti-WHs and anti-WHe antibodies) in a subset of animals	Transient inc. in: − ALT, AST, and SDH	Neutropenia/Thrombocytopenia (reversible)	[82]
		120 mg/kg (po, QOD) + ETV (0.1 mg/kg, po, QD)	6	14/18	7.93	4.68	2.37		Sustained red. in: − WHV RI-DNA and cccDNA, − surface and pg RNAs Transient inc. in: − ALT, AST, and SDH − liver inflammation		
TLR8 agonist	GS-9688/Selgantolimod	1–3 mg/kg (po, QW)	10	8/24	Up to >5.0	Undetectable/Loss	-	SVR/Functional cure (seroconversion to anti-WHs antibodies) in a subset of animals	Sustained red. in: − WHV RI-DNA and cccDNA, − surface and pg RNAs Transient inc. in: − AST and SDH − liver inflammation	Thrombocytopenia in one animal (reversible)	[83]
TLR9 agonist	CpG 21798	4 mg/kg (sc, QW) + ETV (0.5 mg/kg, po, QD)	4	16/12	Undetectable	Undetectable/Loss	-	Transient (delayed viral relapse compared to control)	Transient inc. in: − ALT and AST	None	[84]
	AIC649	10^9^ particles/animal (iv then im, BIW) + ETV (0.2 mg/kg, po, QD)	5	21/-	7.57	4.05	2.46	SVR after ETV withdrawal (seroconversion to anti-WHs and anti-WHe antibodies) in a subset of animals	Sustained red. in: − WHV RI-DNA and cccDNA − surface and pg RNAs Transient inc. in: − ALT, AST, and SDH	None	[85]
RIG-I/NOD2 agonist	SB 9200/Inarigivir	30 mg/kg (po, QD) followed by ETV (0.5 mg/kg, po, QD)	5	16/8	6.4	3.3	-	Transient (delayed viral relapse compared to reversed treatment sequence of ETV followed by SB 9200)	Transient red. in: − WHV RI-DNA and cccDNA, − surface and pg RNAs − liver inflammation Transient inc. in: − AST and SDH	None	[86]
Checkpoint inhibitor	Anti-PD-L1 monoclonal antibody wc6D5	15 mg/kg (iv, every 3rd or 4th day over 10 days) + ETV (0.1 mg/kg, po, QD)	11	12/10	Reduced/Undetectable	Undetectable/Loss	Reduced/Undetectable	SVR in a subset of animals	-	None	[87]
Therapeutic vaccine	DNA-based vaccine encoding WHcAg and WHsAg + Anti-PD-L1 polyclonal antibody	Plasmid DNA (500 µL, im, QW) + Anti-PD-L1 (25 mg/kg, iv, QOD) + ETV (0.2 and 1.5 mg/animal, sc, QD and QW)	3	28/14	Undetectable	Reduced/Undetectable	-	SVR/Functional cure (seroconversion to anti-WHs antibodies) in a subset of animals	Sustained red. in: − WHV RI-DNA and cccDNA	None	[88]

Abbreviations: AAV, adeno-associated virus; AIC649, TLR9 agonist from AiCuris; ALT, alanine transaminase; anti-PD-L1, antibody against PD-L1; anti-WHe, antibodies against WHeAg; anti-WHs, antibodies against WHsAg; APR002, TLR7 agonist from Apros Therapeutics; AST, aspartate aminotransferase; BIW, twice a week; cccDNA, covalently closed circular DNA; CpG 21798, TLR9 agonist from Pfizer; ETV, entecavir; GS-9620, TLR7 agonist from Gilead Sciences; GS-9688, TLR8 agonist from Gilead Sciences; im, intramuscular; inc., increase; iv, intravenous; log, logarithmic; NOD2, nucleotide binding oligomerization domain containing 2; ODU, optical density unit; PD-L1, ligand 1 of programmed cell death protein 1; pg, pre-genomic; po, oral; QD, every day; QOD, every other day; QW, every week; QOW, every other week; red., reduction; RG7854, TLR7 agonist from F. Hoffmann-La Roche; RI-DNA, replicative intermediate DNA; RIG-I, retinoic acid-inducible gene I; SB 9200, RIG-I/NOD2 agonist from Spring Bank Pharmaceuticals; SDH, sorbitol dehydrogenase; sc, subcutaneous; SVR, sustained antiviral response; TIW, three times a week; TLR, toll-like receptor; vg, viral genomes; wc6D5, a monoclonal antibody against woodchuck PD-L1 from Bristol Myers Squibb; WHcAg, WHV core antigen; WHeAg, WHV e antigen; WHsAg, WHV surface antigen; WHV, woodchuck hepatitis virus; wIFN-α5, recombinant woodchuck IFN-α5.
